# Genome-Inferred Correspondence between Phylogeny and Metabolic Traits in the Wild *Drosophila* Gut Microbiome

**DOI:** 10.1093/gbe/evab127

**Published:** 2021-06-03

**Authors:** John G McMullen, Eduardo Bueno, Frances Blow, Angela E Douglas

**Affiliations:** 1 Department of Entomology, Cornell University, Ithaca, New York, USA; 2 Department of Molecular Biology and Genetics, Cornell University, Ithaca, New York, USA

**Keywords:** Rhodospirillales, Lactobacillales, Enterobacterales, functional redundancy, bacterial metabolism, comparative genomics

## Abstract

Annotated genome sequences provide valuable insight into the functional capabilities of members of microbial communities. Nevertheless, most studies on the microbiome in animal guts use metagenomic data, hampering the assignment of genes to specific microbial taxa. Here, we make use of the readily culturable bacterial communities in the gut of the fruit fly *Drosophila melanogaster* to obtain draft genome sequences for 96 isolates from wild flies. These include 81 new de novo assembled genomes, assigned to three orders (Enterobacterales, Lactobacillales, and Rhodospirillales) with 80% of strains identified to species level using average nucleotide identity and phylogenomic reconstruction. Based on annotations by the RAST pipeline, among-isolate variation in metabolic function partitioned strongly by bacterial order, particularly by amino acid metabolism (Rhodospirillales), fermentation, and nucleotide metabolism (Lactobacillales) and arginine, urea, and polyamine metabolism (Enterobacterales). Seven bacterial species, comprising 2–3 species in each order, were well-represented among the isolates and included ≥5 strains, permitting analysis of metabolic functions in the accessory genome (i.e., genes not present in every strain). Overall, the metabolic function in the accessory genome partitioned by bacterial order. Two species, *Gluconobacter cerinus* (Rhodospirillales) and *Lactiplantibacillus plantarum* (Lactobacillales) had large accessory genomes, and metabolic functions were dominated by amino acid metabolism (*G. cerinus*) and carbohydrate metabolism (*La. plantarum*). The patterns of variation in metabolic capabilities at multiple phylogenetic scales provide the basis for future studies of the ecological and evolutionary processes shaping the diversity of microorganisms associated with natural populations of *Drosophila*.

## Introduction

Significance

The metabolic capability of microorganisms can be inferred from genome sequence data but metagenomics and related -omics methods widely used to study complex microbial communities, including microbiomes in animal guts, cannot assign specific metabolic functions to specific taxa with certainty. Our analysis of the genome sequence of 96 bacterial isolates from the gut microbiome of *Drosophila* fruit flies identified considerable metabolic variation at multiple taxonomic levels, ranging from substantial among-order differences to strain-level variation for several species. The assignment, in this study, of function to taxon for members of a complex gut microbiome provides the basis for future studies on ecology and evolution of bacterial metabolism in gut microbiomes.

Animal gut microbiomes are complex assemblages of microorganisms which mediate diverse functions that impact host physiology, behavior, and fitness (Nicholson et al. 2012; Sommer and Bäckhed 2013; Huang et al. 2015; Rolhion and Chassaing 2016; Thaiss et al. 2016; Read and Holmes 2017; Qiao et al. 2019; Turkiewicz et al. 2019). Most interactions between the microbiome and the animal host are based on the metabolic capabilities of microbiome members, with traits ranging from degradation and fermentation of host-inaccessible substrates to synthesis of key nutrients for the host, detoxification of harmful dietary constituents and recycling of metabolic waste products, and effects on host signaling pathways (Hooper et al. 2002; Engel and Moran 2013; Ankrah and Douglas 2018). Investigation of the relationship between traits and taxonomic identity of gut microorganisms has shown that many metabolic traits are functionally redundant and can be shared by closely and distantly related microbiome members (Heintz-Buschart and Wilmes 2018; Louca et al. 2018). This finding is largely based on metagenomic studies, where the taxonomic composition of the microbiome is uncontrolled and variable (Huttenhower et al. 2012; Lozupone et al. 2012).

Functional redundancy can ensure sustained function (also known as ecosystem resilience) of the gut microbiome during perturbations that reduce the abundance or function of specific taxa and alter the overall microbiome composition (Allison and Martiny 2008; Heintz-Buschart and Wilmes 2018). Evolutionary changes, which can occur within ecological timeframes, can also affect the relationship between taxonomy and function. In particular, phylogenetically divergent taxa may share a metabolic trait by gain of function through horizontal gene transfer (HGT), and closely-related taxa may differ in functional traits by differential gene deletions and by functional divergence of a recently duplicated gene (Louca et al. 2018). Two examples illustrate these processes. The first is the bile salt hydrolase gene, which is involved in lipid homeostasis and antimicrobial effects. This gene is widespread across bacterial taxa in the human microbiome (most prevalent among the Firmicutes) with evidence of HGT events among different lactobacilli and *Listeria monocytogenes* (Jones et al. 2008; Kumar et al. 2012; Chand et al. 2017). Secondly, in the honey bee gut microbiome, the distribution of a glucoside hydrolase gene family (genes involved in degradation of hemicellulose in pollen) in *Bifidobacterium* spp. is the result of gene duplication and deletion events (Zheng et al. 2019).

The apparent ubiquity of functional redundancy, however, is open to question. Functional composition analyses often rely on broad metabolic annotations that can encompass multiple pathways (Langille 2018). These methods can fail to detect biologically important differences in metabolic function of gene families, as demonstrated, for example, in Proteobacteria of the human gut microbiome (Bradley and Pollard 2017). Compounding these problems, within-species variation in metabolic function can be widespread, such that metabolic traits important to the host are displayed by only a subset of strains or are mediated by pathways distributed across two or more different strains (Douglas 2020). For example, *Bifidobacterium longum*, a member of the microbiome of the human infant, has a large accessory genome with variable incidence of genes involved in transport and degradation of human milk oligosaccharides, implicating some, but not all, strains of this species as important to human milk metabolism (Vatanen et al. 2019). Intraspecific variation requires identification of not only the pangenome (i.e., total genetic capabilities) of a species, but also how the functional traits are distributed across different strains (Tettelin et al. 2005; Brockhurst et al. 2019; Van Rossum et al. 2020).

The goal of this study was to investigate how primary metabolism functions of a gut microbiome map onto bacterial phylogeny. We used the gut microbiome of *Drosophila melanogaster* for this analysis because, unlike the microbiome of many animals, most of the *Drosophila-*associated bacteria are readily culturable (Douglas 2019). Relative to metagenome-assembled genomes, genome sequences of the individual bacterial isolates enable higher quality assembly and increased resolution of phylogenomic patterns (Van Rossum et al. 2020). More generally, *Drosophila* is a fast-emerging system to investigate ecological and evolutionary questions regarding animal-associated microbiomes (Broderick and Lemaitre 2012; Erkosar et al. 2013; Wong et al. 2016; Douglas 2019) and there are indications that, as for the mammalian gut microbiome, the *Drosophila* metagenome displays incongruence between functional traits and taxonomic composition (Newell et al. 2014; Petkau et al. 2016; Adair et al. 2018; Consuegra et al. 2020; Kang and Douglas 2020). However, the relationship between taxonomy and distribution of traits has not been robustly tested.

For our analysis, we focused on bacterial taxa isolated from natural populations of *Drosophila*, which are associated with rotting fruits (Markow 2015). The gut microbiome of wild *Drosophila* is dominated by members of the bacterial orders Enterobacterales, Lactobacillales, and Rhodospirillales, although the relative abundance of the different taxa varies among individuals and collections (Chandler et al. 2011; Adair et al. 2018; Walters et al. 2020; Wang et al. 2020). Long-term laboratory cultures of *Drosophila* were not used because their gut microbiome is of low diversity (Cox and Gilmore 2007; Staubach et al. 2013; Wong et al. 2013; Obadia et al. 2018) and can be functionally different from wild populations (Winans et al. 2017; Bost et al. 2018). The great majority of published studies on the genome sequences of *Drosophila* gut microorganisms have concerned bacterial taxa derived from laboratory lines (Broderick and Lemaitre 2012; Matos and Leulier 2014) with few sequences available from field isolates (table 1). Therefore, this study was initiated by the isolation of bacteria from field-collected *Drosophila*. In total, we isolated and sequenced the genomes of 81 bacterial strains associated with wild *Drosophila*. We performed comparisons of metabolic traits among all field-isolated strains, and then examined the metabolic pangenomes of prevalent species to assess the scale of within-species variation. Within this panel of bacteria, the three bacterial orders were strongly differentiated by primary metabolic functions, and a subset of species also displayed strain-level variation in metabolism-related genes. The taxonomically variable traits include functions likely to be adaptive for utilization of the sugar-rich rotting fruit environment and are predicted to influence *Drosophila* physiology and performance.

**Table 1:** Bacterial Strains Used in Comparative Genomics Analyses

Order	Family	Genus	Species (Strain ID)	No. Strains Sequenced (no. flies)	Publicly Available Strains
Enterobacterales	Enterobacteriaceae	*Citrobacter*	sp. (C)	1 (1)	
		*Enterobacter*	*asburiae* (Ea)	1 (1)	
			*ludwigii* (El)	1 (1)	
			*mori* (Em)	1 (1)	
			sp. (E)	1 (1)	
		*Klebsiella*	*michiganensis* (Km)	1 (1)	
			*variicola* (Kv)	1 (1)	
	Erwiniaceae	*Pantoea*	*dispersa* (PAd)	2 (1)	
			sp. (PA)	1 (1)	
		***Tatumella***	**sp. #1 (T)**	**6 (6)**	
			sp. #2 (T)	1 (1)	
	Morganellaceae	***Providencia***	*alcalifaciens* (PRa)		1^b^
			*burhodogranariea* (PRb)		1^b^
			***rettgeri* (PRr)**	**4 (4)**	**1b**
			*sneebia* (PRs)		1^b^
			sp. (PR)	3 (3)	
	Yersiniaceae	*Nissabacter*	*archeti* (Na)	1 (1)	
		*Serratia*	*rubidaea* (Sr)	1 (1)	
Lactobacillales	Lactobacillaceae	*Lacticaseibacillusa*	*paracasei* (LApa)	1 (1)	1^c^
		***Lactiplantibacillus^a^***	***plantarum* (LApl)**	**5 (5)**	1^d^
		*Leuconostoc*	*citreum* (LEc)		1^e^
			*mesenteroides* (LEm)	1 (1)	
			*pseudomesenteroides* (LEp)	1 (1)	
			*suionicum* (LEs)	1 (1)	
		***Levilactobacillus^a^***	***brevis* (LAb)**	**5 (5)**	
		*Weissella*	*cibaria* (Wc)		1^f^
			*minor* (Wm)	1 (1)	
	Streptococcaceae	*Lactococcus*	*lactis* (Ll)		1^g^
Rhodospirillales	Acetobacteraceae	***Acetobacter***	*cibinongensis* (Ac)		1^h^
			*indonesiensis* (Ai)		1^h^
			*okinawensis* (Aok)	2 (1)	
			*orientalis* (Aor)		2^h^
			*persici* (Ap)	3 (2)	
			***thailandicus* (Ath)**	**4 (4)**	**1h**
			*tropicalis* (Atr)		1^h^
		***Gluconobacter***	*albidus* (Ga)	1 (1)	
			***cerinus* (G8c)**	**13 (5)**	
			*japonicus* (Gj)	1 (1)	
			***kondonii* (Gk)**	**6 (5)**	
			sp. #1 (G)	3 (2)	
			sp. #2 (G)	1 (1)	
			*sphaericus* (Gs)	3 (2)	
			*wancherniae* (Gw)	3 (1)	

Note.—Prevalent species (detected in four or more flies and represented by >4 strains in our data set) used for pangenome analyses are in bold.

a Genus formally known as *Lactobacillus.*

b Galac and Lazzaro (2012).

c Hammer et al. (2017).

d Petkau et al. (2016).

e Wright et al. (2017).

f Ricks et al. (2017).

g Chaston et al. (2014).

h Winans et al. (2017).

## Results

### Sequencing and Characterization of Bacterial Genomes

**Fig. 1.**
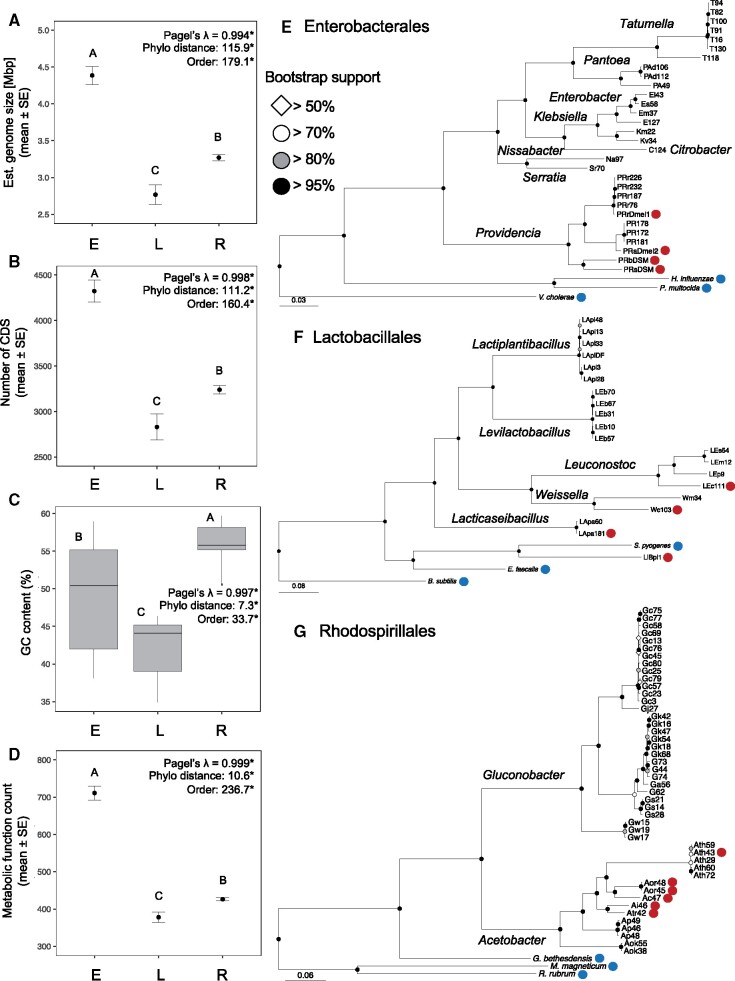
Genomic features and phylogenomic analysis of *Drosophila*-associated bacteria. (*A*) Estimated genome size, (*B*) number of CDS (coding sequences), (*C*) GC content, and (*D*) RAST metabolic function counts by bacterial order. (*E* and *F*) Phylogenomic reconstruction for (*E*) Enterobacterales, (*F*) Lactobacillales, and (*G*) Rhodospirillales. For all genomic features, the raw means and standard error are displayed, except for box plots used in panel (*C*) to show GC content. Pagel’s λ and *F* or χ^2^ statistics for model predictors (order and phylogenetic distance) are displayed for each panel (residual df = 92). *All *P* values are <0.01. Phylogenetic distance is calculated from branch lengths of phylogenomic reconstruction. Letters represent statistical grouping from post hoc Tukey’s test. Phylogenomic analysis is based on the concatenated sequence (length = 13,238 amino acids) of 52 genes (details in supplementary table S7, Supplementary Material online and alignment in supplementary data set S4, Supplementary Material online), and the genus names of *Drosophila*-associated strains studied are displayed near the nodes. The circles near the tips of the phylogenies indicate genomes that are previously published (red, *Drosophila*-associated; blue, references). Dendrograms are scaled to amino acid divergence. Data for panels (*A*–*D*) are provided in supplementary data set S1, Supplementary Material online. E, Enterobacterales; L, Lactobacillales; R, Rhodospirillales.

We assessed whether primary metabolism functions found in gut bacterial microbiome members of wild *Drosophila* can be mapped onto bacterial taxonomy. First, we characterized the genomic features of the strains from each bacterial order. Given that few bacterial species associated with wild *Drosophila* have been isolated and sequenced previously, we collected and sequenced 81 newly isolated strains that are members of the three dominant bacterial orders (i.e., Enterobacterales, Lactobacillales, and Rhodospirillales) found within the fly gut to complement the 15 genomes currently available (table 1 and supplementary table S1, Supplementary Material online). Genome features (genome size, number of coding sequences [CDS], and GC content) of all newly sequenced taxa (supplementary table S1*A*, Supplementary Material online) were similar to publicly available species. The estimated genome sizes of the strains sequenced ranged from 1.8 to 5.8 Mb with 1,879–5,983 CDS and GC content of 37–60% for the 96 *Drosophila-*associated strains. Average coverage (i.e., sequence depth) of genomes ranged from 53× to 1,390× (supplementary table S1*A* and data set S1, Supplementary Material online). The number of metabolic functions per genome annotated by RAST (i.e., RAST role or encoded gene function) ranged from 286 to 968 (supplementary data set S1, Supplementary Material online). Comparisons of genomic features indicated that all measures significantly differed by bacterial order (fig. 1*A*–*D*). In addition, phylogenetic signal was assessed among the 96 *Drosophila*-associated strains to determine whether genome characteristics were shared among closely related taxa. Using two complementary methods, 1) Pagel’s λ and 2) patristic distance (based on phylogenomic analysis) as a covariate in ANOVA and logistic regression analyses, all four genomic features scored had statistically significant results for both tests (fig. 1*A*–*D*), indicating that closely related taxa tend to have similar genome characteristics.

The taxonomy of the newly isolated bacterial strains was characterized by two methods: genome comparisons of average nucleotide identity (ANI) to genomes of type specimens, and BlastN search of genome extracted 16S rRNA gene against the nonredundant NCBI database. Based on ANI scores, 80% of the strains were identified to the species level (supplementary table S1*A*, Supplementary Material online). The remainder of the strains was identified to the genus level using 16S rRNA gene sequence where no close ANI match was available (supplementary table S1*A*, Supplementary Material online). In addition, a previously sequenced genome *Acetobacter* sp. DmW-043 (Winans et al. 2017) was identified as *Acetobacter thailandicus* (98.9% ANI to *A. thailandicus* LMG 30826, accession: GCA_011516655, which was not available at the time of publishing this genome sequence). A phylogenomic analysis of 52 single-copy orthologs supported the ANI species boundaries with strong bootstrap node support (generally >95%, although some of the Rhodospirillales species had node support >70%; fig. 1*E*–*G* and supplementary fig. S1, Supplementary Material online). All species and genera formed monophyletic clades. In addition, the formerly paraphyletic genus *Lactobacillus*, which was recently reclassified into 25 different genera, matched the results from larger phylogenomic analyses of this group with *Leuconostoc* and *Weissella* spp. embedded among *Lacticaseibacillus, Lactiplantibacillus*, and *Levilactobacillus* spp. (Salvetti et al. 2018; Zheng et al. 2020). Similarly, the evolutionary relationships between taxa of the Enterobacterales and Rhodospirillales were consistent with published data sets containing additional species from each order (Matsutani et al. 2011; Adeolu et al. 2016; Baek et al. 2020; Yukphan et al. 2020).

### Association of 16S rRNA Gene with Phylogenomic Relationships

As 16S rRNA gene amplicon sequencing is widely used in taxonomic surveys for microbiome studies, we investigated how well 16S sequence predicted species identity and phylogenomic relationships of the strains used in this study. In the BLAST top matches with 16S rRNA genes, 77% of the strains had more than one species match (supplementary table S1*A*, Supplementary Material online). Similarly, many of the 16S rRNA genes of bacterial strains identified to different species yielded sequence identity matches ≥97%, which is the general threshold for species boundaries in bacteria. This indicates that sequence identity scores were not appropriate to resolve species boundaries for these taxa. This applied especially to *Gluconobacter* and *Leuconostoc* spp. and many of the Enterobacterales strains (supplementary table S2, Supplementary Material online), which is consistent with published data of these taxa (Matsutani et al. 2011; Adeolu et al. 2016; Jeon et al. 2017), and indicated that the 16S rRNA gene does not always infer species identity reliably. Phylogenetic analysis of 16S rRNA genes tended to have lower bootstrap support than the phylogenomic analysis (supplementary fig. S2, Supplementary Material online). Many of the species clusters identified by phylogenomics were evident in the 16S phylogeny, but some of the Enterobacteriaceae and *Gluconobacter* spp. were mis-identified as polyphyletic (supplementary fig. S2, Supplementary Material online).

**Fig. 2.**
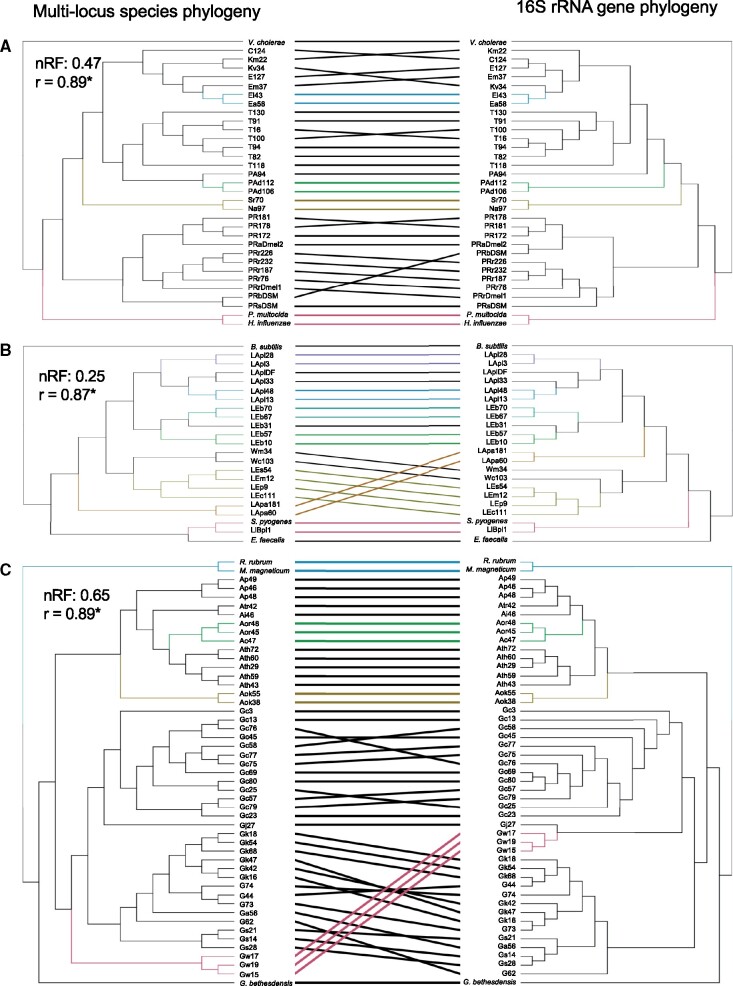
Relationship between multilocus and 16S rRNA gene phylogeny. Tanglegrams for (*A*) Enterobacterales. (*B*) Lactobacillales. (*C*) Rhodospirillales. Normalized Robinson–Foulds (nRF) indices and Mantel test correlations are displayed for each order. Subtrees with the same topologies between each dendrogram are colored. *P = 0.001.

Two complementary methods were implemented to compare congruence between the phylogenomic analysis and 16S rRNA phylogeny. First, normalized Robinson–Foulds index was used to compare dendrogram topologies, which indicated that 16S rRNA gene phylogeny had the best correspondence with Lactobacillales and weakest association with the Rhodospirillales (fig. 2). Second, a Mantel test was implemented to correlate the cophenetic distances between all taxa of each dendrogram. All three bacterial orders displayed strong, statistically significant correlation, indicating that 16S rRNA gene phylogeny retains much of the overall higher order taxonomic placement of species relationships found in the phylogenomic analysis, but fails to discriminate some taxonomic distinctions at finer resolutions between and within species; its shortcomings are considered further in the Discussion section.

### Correspondence between Metabolic Traits and Phylogeny

**Fig. 3.**
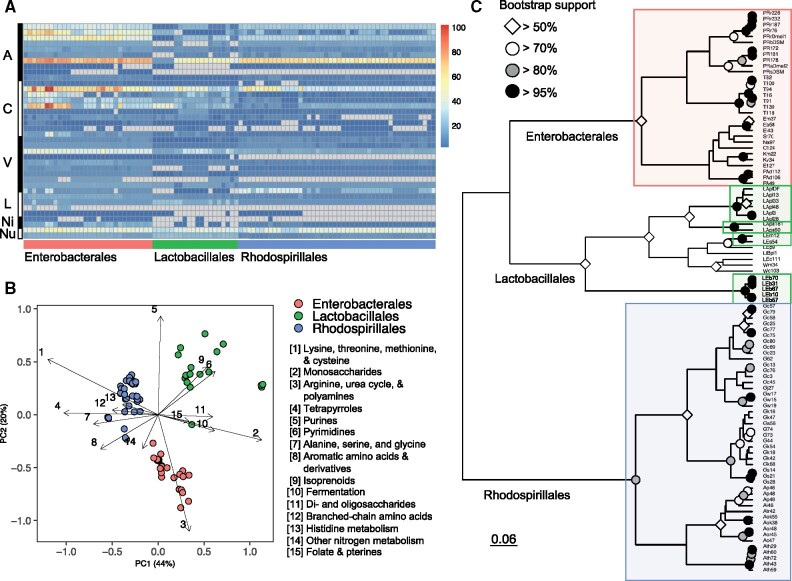
Taxonomic correspondence with encoded metabolic functions. (*A*) Heatmap of raw function counts in RAST subcategories displayed by bacterial order. Rows and columns are organized by alphabetical order for RAST categories and bacterial taxonomy. Gray cells in heatmap indicate function is absent in genome. RAST subcategories are grouped by categories: A, amino acids and derivatives; C, Carbohydrates; V, cofactors, vitamins, prosthetic groups, and pigments; L, fatty acids, lipids, and isoprenoids; Ni, nitrogen metabolism; Nu, nucleosides and nucleotides. (*B*) Principal coordinates analysis (PCoA) of relative counts for RAST subcategories with Bray–Curtis dissimilarity matrix. Arrows indicate the loading subcategories (top 15 displayed) and the percent variance explained for each axis is displayed. (*C*) Hierarchical cluster of relative counts for RAST subcategories. Significant clusters are boxed and colored by bacterial order. Cophenetic distance scale for Ward’s linkage is displayed. RAST function counts are provided in supplementary data set S3, Supplementary Material online.

Bacterial traits (in this case encoded metabolic gene functions) were grouped by the 38 RAST subcategories related to primary metabolism to infer correspondence between bacterial phylogeny and distributions of metabolic functions. Despite some variability in counts between genomes from each order, the taxa belonging to the order Enterobacterales tended to have more functions related to amino acid, carbohydrate, and vitamin metabolism than Lactobacillales and Rhodosprillales, whereas functions involved in lipid, nitrogen, and nucleotide metabolism generally had similar counts across all taxa (fig. 3*A*). The expanded range of functions in the Enterobacterales is likely linked to the relatively large genome size and CDSs in these bacteria (fig. 1).

Principal coordinates analysis (PCoA) was applied to visualize the relationship between taxonomy and metabolic potential using relative counts to normalize the data (fig. 3*B*). On the first axis, the three bacterial orders were distinctly separated, whereas on the second axis, the Enterobacterales were separated from the other two orders. PERMANOVA indicated a large effect by bacterial order on metabolic trait groupings (*F*_2,93_ = 61.72, *P* = 0.001, *R*^2^ = 0.57) and a pairwise PERMANOVA analysis revealed that all three orders were significantly different from each other (*q* < 0.05) (supplementary table S3*A*, Supplementary Material online). In addition, each of the clusters separated by genus-level taxonomy, apart from some mixing between *Providencia* and *Tatumella* spp., further indicating metabolic differentiation by taxonomy (supplementary fig. S3, Supplementary Material online).

The top 15 loadings from PCoA were displayed to identify RAST subcategories that were associated with each bacterial order (fig. 3*B*). Generally, each order was associated with different metabolic functions; the Rhodospirillales were driven by amino acid metabolism, whereas the Lactobacillales were associated with carbohydrate, nucleotide, and lipid metabolism and Enterobacterales were influenced by arginine, urea cycle, and polyamine metabolism. Both the Lactobacillales and Rhodospirillales were associated with functions related to vitamin and cofactor metabolism. Lastly, the Rhodospirillales may share some of the nitrogen metabolism functions with Enterobacterales, as it relates to different organic and inorganic nitrogen metabolic pathways (subcategory contains ammonia fixation, allantoin utilization, and amidase subsystems).

The metabolic functions of each strain were further clustered using an agglomerative hierarchical method and significant clusters were identified using a multiscale bootstrap resampling approach with 10,000 replicates (fig. 3*C*). The three orders separated from one another with >50% bootstrap probability support. The Enterobacterales and Rhodospirillales bacteria formed two significant clusters with almost all of the genera grouped together for each order (except some of the *Gluconobacter* spp.). The Lactobacillales formed three significant clusters by species (*Lacticaseibacillus paracasei, Lactiplantibacillus plantarum*, and *Levilactobacillus brevis*), as well as another *Leuconostoc* spp. cluster (fig. 3*C*), which all had >95% bootstrap probability support. The remainder of the Lactobacillales species only had single strain representatives, likely influencing the lack of clusters.

To further understand the relationship between phylogeny and distribution of metabolism functions, the hierarchical cluster was correlated with phylogenomic and 16S rRNA gene dendrograms using normalized Robinson–Foulds index (nRF) and Mantel test. Overall topologies of dendrograms were moderately associated between phylogeny and metabolic traits (phylogenomic analysis: nRF = 0.53, *r* = 0.79, *P* = 0.001; 16S rRNA gene analysis: nRF = 0.62, *r* = 0.68, *P* = 0.001), with the best congruence found when associating function with phylogenomic analysis (likely driven by the congruence of the Enterobacterales members with nRF of 0.37 compared with Lactobacillales and Rhodospirillales nRF scores of 0.53 and 0.65, respectively) (supplementary fig. S4, Supplementary Material online). Results from Mantel test comparing cophenetic distances of each phylogeny with Bray–Curtis dissimilarities supported this finding with a 1.2× increase in correlation statistic when using the phylogenomic reconstruction compared with the 16S rRNA gene phylogeny, suggesting that the increased resolution of the species tree amplified the phylogenetic signal for overall distribution of metabolic traits. Further inspection of the tanglegrams indicated that few strains had overlapping topologies between dendrograms (i.e., displayed the same node-edge relationships), further supporting weak to moderate congruence between dendrograms found for each order (supplementary fig. S4, Supplementary Material online).

As a complementary analysis, orthogroups were identified between all 96 taxa to determine whether a finer resolution at the gene family incidence level (i.e., presence–absence of orthogroups) would reflect the functional relationships observed based on RAST annotations. A PCoA was used to visualize the 13,170 orthogroups with genes from at least three genomes (supplementary table S4, Supplementary Material online) using a Jaccard similarity coefficient to determine whether there are differences by bacteria order based on orthogroup assignments. All three bacterial orders clustered away from one another, with Rhodospirillales separating on the first axis away from the Enterobacterales and Lactobacillales and all three orders distinctly separating on the second axis (supplementary fig. S5, Supplementary Material online). Orthogroup composition of each genome significantly differed by order (PERMANOVA: *F*_2,93_ = 63.2, *R*^2^ = 0.58, *P* = 0.001, supplementary table S3*B*, Supplementary Material online). Of the total orthogroups identified, 8% were involved in metabolism-related functions (defined by RAST annotations). These 1,055 orthogroups were extracted and subjected to the same analysis, resulting in a similar finding that all three bacterial orders are distinct on both PCoA axes (supplementary fig. S5, Supplementary Material online), with PERMANOVA support (*F*_2,93_ = 106.5, *R*^2^ = 0.7, *P* = 0.001, supplementary table S3*C*, Supplementary Material online). In addition, a Procrustean randomization test indicated that the orientation of metabolism-related orthogroups was highly correlated with the overall relationship among all orthogroups (*m*^2^ = 0.008, *r* = 0.996, *P* = 0.001).

### Variation in Metabolism Genes of Prevalent Species

To extend our analysis of metabolic variation among the *Drosophila*-associated bacteria, we focused on seven species, which we termed “prevalent” by the criteria that they were isolated from at least four flies and were represented by >4 strains (table 1). Based on the incongruence observed between metabolic function and taxonomy (supplementary fig. S4, Supplementary Material online), we further analyzed these taxa to identify differences among and within species to better understand functional variation and redundancy at finer phylogenetic scales. Specifically, these taxa provide the opportunity to define the distribution of metabolic traits from a pangenomic perspective, including comparisons in orthogroup membership between species and identification of among-strain variation, that is, enriched functions in the accessory genome, with taxa that are well-represented within the data set.

**Fig. 4.**
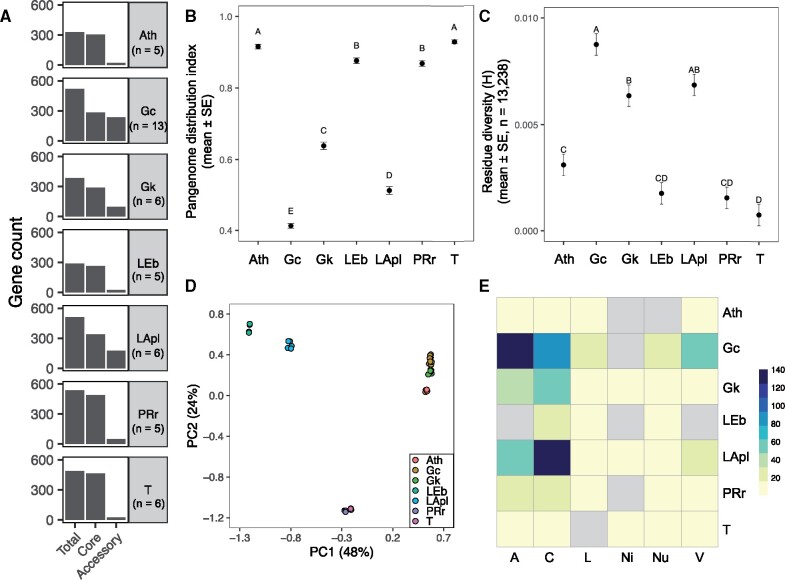
Metabolic pangenome analysis of prevalent species. (*A*) Distribution of metabolism genes in pangenome of each species with number of strains listed below each taxon identifier. (*B*) Relative pangenome distribution by species. (*C*) Strain diversity by species using Shannon’s entropy score. (*D*) PCoA of orthogroup composition among species. (*E*) Heatmap of function counts (20–140) in accessory genome by species. In panels (*B*) and (*C*), estimated marginal means and standard error are plotted from each model with letters from post hoc Tukey’s test representing statistical groups. Percent variation explained among significantly different orthogroups is shown for each axis in panel (*D*). Gray cells in heatmap of panel (*E*) indicate function is absent in accessory genome. Species identifiers: Ath, *Acetobacter thailandicus*; Gc, *Gluconobacter cerinus*; Gk, *G. kondonii*; LEb, *Levilactobacillus brevis*; LApl, *Lactiplantibacillus plantarum*; PRr, *Providenica rettgeri*; T, *Tatumella* sp. RAST categories: A, amino acids and derivatives; C, Carbohydrates; V, cofactors, vitamins, prosthetic groups, and pigments; L, fatty acids, lipids, and isoprenoids; Ni, nitrogen metabolism; Nu, nucleosides and nucleotides. Data are provided in supplementary data set S2 and table S5, Supplementary Material online.

A pangenome analysis was performed using Roary to identify single-copy orthologous genes encoding metabolic functions found within each of the seven species and to define the distribution of genes found in the metabolic pangenome. Across the seven species, the total pangenome ranged from 287 to 538 metabolism-related genes (based on RAST annotations) with core genome size of 264–488 genes and accessory genome size of strains ranging from 0 to 102 genes (fig. 4*A* and supplementary data set S2, Supplementary Material online). A distribution index was generated to compare the relative sizes of the metabolic pangenome with values close to 1 indicating a small accessory genome with few genes per strain; values closer to 0 indicated high strain diversity with equal numbers of genes in the core and accessory genome for a strain. A beta regression of indices for each species indicated significant differences by species (likelihood ratio test: χ^2^_6_ = 200.2, *P* < 2.2 × 10^−16^) with no specific statistical similarities between species with similar taxonomy, although both *Providenciarettgeri* and *Tatumella* sp. (Enterobacterales) had small metabolic accessory genomes (fig. 4*B*). The relatively large accessory genome sizes of *Gluconobactercerinus, G. kondonii*, and *La. plantarum* correlated with increased residue diversity (index for measuring average strain diversity within species using the amino acid alignments from the phylogenomic analysis) by correlation analysis (Pearson’s product-moment correlation: *r* = 0.9, *P* = 0.003) (fig. 4*C* and supplementary fig. S6, Supplementary Material online). This result gives confidence that the identified accessory genome has a biological basis, and is not a sequencing artifact; accessory genome size is predicted to increase positively with strain diversity. Additionally, nucleotide diversity based on 16S rRNA genes among strains was also assessed and no significant relationships were found, indicating the increased resolution of the phylogenomic analysis was required to score strain diversity between species (supplementary fig. S6, Supplementary Material online).

The contribution of between- and within-species differences to variation in metabolic traits was investigated using two methods. In the first approach, the metabolic traits (defined by RAST annotation) between species were compared using Fisher’s exact test on metabolism orthogroup incidence (i.e., orthogroup presence–absence by species; minimum threshold of three genomes represented per orthogroup) (supplementary table S5*A*, Supplementary Material online). After *P* value adjustment, 597 of the 717 orthogroups were significantly different between species (*q* < 0.05) (supplementary table S5*A*, Supplementary Material online). A PCoA with a Jaccard similarity coefficient was used to visualize how species separated by significant orthogroups to summarize these differences. All seven species formed distinct taxonomic clusters (PERMANOVA: *F*_6,39_ = 775.23, *R*^2^ = 0.99, *P* = 0.001, supplementary table S3*D*, Supplementary Material online) with bacteria separating by order on the first axis and Lactobacillales and Rhodospirillales separated from the Enterobacterales strains on the second axis (fig. 4*D*). The top RAST category was assigned to each orthogroup and associated with each PCoA axis, indicating Rhodospirillales were enriched in amino acid, nucleotide, and vitamin metabolism in PC1, whereas Enterobacterales were enriched in all metabolism categories except nucleotide metabolism in PC2 (supplementary fig. S7, Supplementary Material online). This analysis of seven species largely recapitulates the analysis of all strains, as displayed in figure 3*B*.

Further investigation into the orthogroup analysis established that the top significant orthogroups from the Fisher’s exact test (supplementary table S5*A*, Supplementary Material online) were primarily involved in carbohydrate metabolism (∼50%) and that most orthogroups were present in the two *Gluconobacter* spp., whereas the other taxa had lower incidence rates across gene families (supplementary table S5*B*, Supplementary Material online). When a given species was a member of a top orthogroup identified, all strains were found to contain at least one gene from this gene family (supplementary table S5*A*, Supplementary Material online). In addition, all of the top orthogroups were present in at least two species, and they were generally not defined by higher order taxonomy (e.g., *Gluconobacter* and *Tatumella* spp. tended to have similar orthogroup functions). Of the top gene functions identified, several were noteworthy for known effects on *Drosophila* physiology. Some of the sugar and sugar derivative dehydrogenases have been implicated as determinants of reduced lipid content in adult flies by incomplete oxidation of external carbohydrates (Chaston et al. 2014). Our analysis (supplementary table S5*B*, Supplementary Material online) also identified a bacterial methionine salvage gene (5-methylthioribose kinase), which bacterial methionine metabolism lowers starvation resistance of *Drosophila* (Judd et al. 2018), and the hydroxymethylpyrimidine ABC transporter involved in the production of thiamine (vitamin B_1_) (supplementary table S5*B*, Supplementary Material online), an important determinant of larval development and survival on low-nutrient diets (Sannino et al. 2018). In addition, several gene functions that aid in the bacterial growth and utilization of *Drosophila* metabolic waste products (Winans et al. 2017; Storelli et al. 2018) were identified (supplementary table S5*B*, Supplementary Material online). Notably, N-acetylglucosamine gene families (the monomer of chitin found in the peritrophic envelope of the insect gut as well as fungal cell walls) and xanthine degradation gene families (part of an Acetobacteraceae uric acid degradation locus, the primary nitrogen waste product of *Drosophila*) were among the bacterial functions found in the top orthogroups identified from the Fisher’s exact test (supplementary table S5*B*, Supplementary Material online). For the latter orthogroup, we further inspected whether the uricase gene was also present in the genomes of the prevalent strains, as it is not a function classified by the RAST subsystem annotations. This gene was present in all genomes of prevalent *Gluconobacter* and *Tatumella* spp. and is part of the orthogroup OG0001450 (supplementary table S4*B*, Supplementary Material online), indicating these taxa may potentially utilize uric acid in the excreta of *Drosophila*.

Our second analysis of variation in metabolic traits across the seven prevalent species identified gene functions enriched in the accessory genome of each species compared with the core genome (supplementary table S6*A–G*, Supplementary Material online), which may indicate adaptive functions that enhance strain fitness. Among the different functional annotation counts in the accessory genome, amino acid metabolism in *G. cerinus* and carbohydrate metabolism in *La. plantarum* were the highest and nitrogen metabolism were low or absent in all seven species (fig. 4*E*). Only seven RAST subsystems were identified as enriched in the accessory genome of four species (*G. kondonii*, *Le. brevis, La. plantarum*, and *P. rettgeri*) after *P* value adjustment for multiple testing (*q* < 0.05) (supplementary table S6*H*, Supplementary Material online). Each species included carbohydrate metabolism gene functions predicted to expand the capacity of the bacteria to utilize and ferment different carbohydrates (potentially glucose, gluconate, fructose, mannose, and trehalose) and the carboxylic acid citrate, which is important for the growth and acid resistance of lactobacilli (Martin et al. 2005). Lipid/carbohydrate metabolism (related to short chain fatty acid butyric acid fermentation) and purine biosynthesis were implicated as enriched in the *P. rettgeri* accessory genome (supplementary table S6*F*, Supplementary Material online). Most of the ortholog functions in *La. plantarum* and *P. rettgeri* were exclusively found in these taxa, whereas the other orthologs of *G. kondonii* and *Le. brevis* were found in the pangenomes of at least one other prevalent species examined (supplementary table S6*H*, Supplementary Material online), indicating that some of the accessory genome functions can be redundant among closely and distantly related taxa.

## Discussion

A robust understanding of the relationship between the taxonomic identity and functional traits of microorganisms is essential for detailed analyses of the ecological and evolutionary processes that shape microbial communities. This relationship is particularly important for the microbial communities in animal guts because microbial function can influence many host traits, but the pattern and scale of the effect of variation in taxonomic composition on microbial function are poorly understood. This study on the comparative genomics of bacteria isolated from the guts of wild *Drosophila* focused on bacterial metabolic traits, which have been implicated in the metabolic health and fitness of animal hosts (McFall-Ngai et al. 2013; Visconti et al. 2019), including *Drosophila* (Chaston et al. 2014; Newell et al. 2014; Bost et al. 2018; Consuegra et al. 2020). Two key results were obtained. First, representatives of the three dominant bacterial orders (Enterobacterales, Lactobacillales, and Rhodospirillales) can be differentiated by key metabolic traits, based on annotations and homology of metabolism-related genes. Second, evidence for within-species variation in metabolic functions was obtained, including functions relevant to utilization of the sugar-rich habitats and interactions with the *Drosophila* host. Here, we consider these two issues in turn.

Our finding that the variation in metabolic function partitions by the three bacterial orders of gut bacteria (fig. 3) reflects the differences in lifestyles of the bacteria. Important for interpretation of these results, these differences relate exclusively to the panel of genomes isolated from *Drosophila* guts, comprising members of just one, two, and four families for Rhodospirillales (five families in total on NCBI), Lactobacillales (five families in total on NCBI), and Enterobacterales (nine families in total on NCBI), respectively (table 1). The diversity of taxa studied are functionally restricted by the conditions in the *Drosophila* gut, including physical instability, hypoxia (but not anoxia), low pH, and immunological defenses (Lemaitre and Miguel-Aliaga 2013; Douglas 2018). A further potential issue is that some taxa in the *Drosophila* gut microbiome may be intractable to cultivation but the magnitude of this difficulty is likely low because the taxa in the genome panel (table 1) match well to the results from cultivation-independent studies on *Drosophila* collected from the same habitats in New York State (Adair et al. 2018; Bost et al. 2018; Kang and Douglas 2020). Further studies are required to assess whether these conclusions apply to flies in other locations.

The key lifestyle features of Acetobacteraceae (Rhodospirillales) relate to their adaptation to high sugar habitats, such as the rotting fruits utilized by *Drosophila* (Lievens et al. 2015). The distinctive metabolic features identified in this study (fig. 3*B*) relate to aerobic fermentation of exogenous sugars via processes dependent on the tetrapyrrole derivative pyrroloquinoline quinone (Matsutani and Yakushi 2018) and the capacity to utilize simple inorganic and organic nitrogenous substrates for the synthesis of amino acids required for protein synthesis and proliferation (Sainz et al. 2017). Similarly, all but one of the Lactobacillales in this study comprised members of the family Lactobacillaceae and have the functional traits of fermentative metabolism, especially of sugars and other organic compounds, including terpenes and nucleotides (Duar et al. 2017). Many of the products from these metabolic pathways are likely to be important for *Drosophila* growth and physiology, as illustrated by the evidence that the amino acids produced by *Acetobacter* may promote *Drosophila* larval development (Consuegra et al. 2020).

On the contrary, the Enterobacterales associated with *Drosophila* are taxonomically and functionally more diverse (fig. 1*D* and *E*). The lifestyles represented by the Enterobacterales in our panel likely include both free-living bacteria associated with the food ingested by the flies and taxa that may be pathogenic to *Drosophila*, for example, some strains of *P. rettgeri* (Galac and Lazzaro 2011; Adair et al. 2018). This metabolic diversity probably accounts for the single metabolic trait (i.e., arginine, urea cycle, and polyamine metabolism pathway) that partitions with the Enterobacterales (fig. 3*B*). Unlike the Acetobacteraceae and Lactobacillaceae, relatively little is known about the dynamics of Enterobacterales and other γ-Proteobacteria in the *Drosophila* gut, beyond the observations that γ-Proteobacteria are generally not detectably beneficial, or can be detrimental, to *Drosophila* (e.g., Galac and Lazzaro 2011; Chaston et al. 2014), and that host filtering processes may limit their abundance in the gut (Wang et al. 2020). The association of Enterobacterales with the urea cycle and polyamine synthesis raises the possibility that the association of these bacteria with *Drosophila* may be facilitated by their capacity to utilize *Drosophila* waste urea as a nitrogen source and to tolerate hostile conditions in the gut via polyamine-mediated stabilization of the genome and membranes. Microbiome-mediated polyamine production has also been implicated in microbiome effects on human health (Tofalo et al. 2019), but the role of this class of metabolites in *Drosophila*-microbe interactions has not been investigated.

The parallel analysis of within-species variation, conducted on seven species with at least five sequenced genomes, provided the opportunity to assess the scale of among-strain genetic and functional variation in metabolism, including metabolic traits with known effects on *Drosophila* nutritional physiology and performance (e.g., Shin et al. 2011; Chaston et al. 2014; Winans et al. 2017; Judd et al. 2018; Kang and Douglas 2020). For this analysis, we used two approaches. First, we compared between-species genetic variation (fig. 4*D*), which was congruent with annotation-based analysis in figure 3*B*. Of the top gene functions found to vary by species, only a few are known to be relevant determinants of *Drosophila* physiology and some were functionally redundant across disparate taxa (supplementary table S4, Supplementary Material online). Several genes involved utilization of *Drosophila* nitrogenous waste products were identified, primarily among *Gluconobacter* spp., and these capabilities may allow the taxa to use host nitrogenous waste for their own growth. The second analysis focused on identifying functions enriched in the accessory genome of each species. Interestingly, the majority of the genes that differed within species related to carbohydrate digestion and fermentation as well as carboxylic acid and short chain fatty acid metabolism. The enrichment of carbohydrate metabolism genes is also supported by published pangenome analyses of *La. plantarum* and *P. rettgeri* (Galac and Lazzaro 2012; Martino et al. 2016). Taken together, the identified gene functions are suggestive of survival in sugar-rich rotting fruit environment that is enriched by the waste products of *Drosophila* larvae and possibly adults (Lievens et al. 2015; Winans et al. 2017; Storelli et al. 2018).

Rotting fruit provide an energy-rich but ephemeral resource colonized by numerous microorganisms. In this environment, there is strong selective pressure to utilize carbon sources due to exploitative competition and the release of toxic metabolic by-products by co-occurring microbes (e.g., citrate lyase gene functions can be involved in acid stress in lactobacilli; Martin et al. 2005). Although we did not sample strains from rotting fruits, various studies indicate that there is frequent cycling of microbes between wild *Drosophila* gut and the external environment (Blum et al. 2013; Inamine et al. 2018; Pais et al. 2018), and that this likely limits taxonomic and functional differentiation between strains in *Drosophila* and the external environment (Winans et al. 2017; Bueno et al. 2019; Wang et al. 2020). A related issue is the taxonomic and functional differences between bacteria in the natural environment and associated with laboratory cultures of *Drosophila.* The limited data available have not identified fixed differences between laboratory-derived bacteria and field isolates, although a higher incidence of genes coding uric acid degradation in laboratory isolates, and of motility genes in wild isolates has been reported in one study of Acetobacteraceae (Winans et al. 2017). Much of the knowledge of microbiome effects on *Drosophila* metabolism has focused on bacteria isolated from laboratory flies (e.g., Shin et al. 2011; Chaston et al. 2014; Newell and Douglas 2014; Consuegra et al. 2020), and future work would benefit from the inclusion of wild-derived bacterial strains.

This study also raises methodological issues. One issue relates to the utility of 16S rRNA gene sequence data for taxonomic identification and inference of functional traits. Our analysis reinforces the conclusion of many previous studies, including research on microbiomes, that 16S data can be insufficiently precise to discriminate functionally different microorganisms because functionally important sequences are gained, lost or modified by mutation more rapidly than 16S sequence change (Koeppel and Wu 2013; Ellegaard and Engel 2016; Lladó Fernández et al. 2019). 16S rRNA gene sequence evolution can also yield phylogenetic patterns that are incongruent with patterns from phylogenomic data, as illustrated for several taxa in figure 2 as well as other bacterial orders (Maayer et al. 2019). Although not explored in this study, other housekeeping genes, for example, *gyrB, rpoB*, have been suggested as alternatives to 16S rRNA gene for amplicon-based microbiome studies (Moeller et al. 2016; Ogier et al. 2019). For these reasons, inferring function from 16S gene surveys (Langille et al. 2013) is less satisfactory than genomic and metagenomic data. A second issue relates to the key limitation of genomic data, that these data provide the genetic capacity for function, and the realized capacity is dictated by gene expression, enzyme activity, and pattern of flux through the metabolic network of individual microbial cells and the microbial community (Heintz-Buschart and Wilmes 2018). In microbiomes, as in other complex microbial communities, the metabolic traits of individual bacterial taxa can be strongly dependent on the identity and metabolic activity of other co-occurring microorganisms, such that the metabolic function of any taxon can be resolved most effectively by a community approach (e.g., Fischer et al. 2017; Douglas 2020; Henriques et al. 2020; McMullen et al. 2020). A final issue relates to the use of draft genomes in comparative genomics analyses. Draft genomes include poorly sequenced regions of the genome (e.g., due to repetitive regions and mobile genetic elements) and can have genes split across contigs (Ricker et al. 2012). For pangenome analyses, these limitations can lead to genes being miscategorized as accessory (i.e., not present in all strains). In the present analysis, many of the genomes were of draft status, and therefore some designations of accessory functions may be inaccurate. Nevertheless, the highly significant positive correlation between strain diversity and the accessory genome size (supplementary fig. S6*B*, Supplementary Material online) indicates strongly that the observed variation in pangenome size has a biological basis.

We conclude by considering how this study informs our understanding of metabolic trait distribution among members of animal gut microbiomes. The taxonomic and functional composition of animal gut microbiomes are influenced by diet, host, and co-occurring microorganisms. By identifying the microorganisms that mediate different functions and their evolutionary relationships, this study provides a basis to understand and predict microbiome functions, which is the foundation for rationally designed routes to manipulate microbiomes for treatment of metabolic disease and application of probiotics (Bauer et al. 2015). The identification of variation in metabolic functions at different phylogenetic scales in this study provides the basis for future studies to determine the ecology and evolution of microbiome functions of *Drosophila* in natural settings.

## Materials and Methods

### Isolation of *Drosophila*-Associated Bacteria

Wild *D. melanogaster* flies were collected from compost bins or other food waste from five domestic kitchens in Ithaca, NY and from a dumpster containing rotting fruits at the Cornell Orchards, Ithaca, NY, from 2015 to 2019 (see supplementary table S1*A*, Supplementary Material online, for collection details). Flies were starved for 1–3 h to allow any food in the gut to be eliminated, and then anesthetized with CO_2_ and sorted by sex (distinguished visually by genitalia morphology) and species following the key of Werner and Jaenike (2017) to obtain *D. melanogaster* adults. Although *D. melanogaster* female flies are indistinguishable from *Drosophila simulans* female flies, we did not detect any *D. simulans* males in our collections and included female flies to enhance our collection in 2015. The flies were washed in sterile phosphate buffered-saline (PBS) (Cold Spring Harbor 2018), and hand-homogenized in 100 μl PBS (except 200 μl for 2019 Lactobacillales collections) with a disposable pestle (Kontes/Kimble-Chase, Vineland, NJ) using aseptic technique. Each homogenate was inoculated onto an agar medium (yeast–peptone–dextrose [YPD] or modified De Man, Rogosa, and Sharpe [mMRS]) and incubated at 30 °C for up to 1 week under aerobic or high CO_2_ conditions by placing a lit candle in a glass jar (Fan and Li 1997) (supplementary table S1*A* and *B*, Supplementary Material online). YPD is a nutrient rich medium that supports the growth of sugar-rich environment microorganisms (Dunitz et al. 2014), whereas mMRS is a more selective medium that promotes the growth of acetic acid bacteria (Rhodospirillales) and lactobacilli (Lactobacillales) associated with *Drosophila* (Newell and Douglas 2014). In 2019, the procedure was modified to enhance the efficacy of isolating lactobacilli, which tend to have low relative abundance in wild fly guts (Chandler et al. 2011; Staubach et al. 2013; Adair et al. 2018; Kang and Douglas 2020). Specifically, the homogenates were allowed to settle for 5–10 min (allowing large microorganisms, e.g., yeasts, to settle) and 75 μl supernatant was inoculated on agar plates. The mMRS medium was also supplemented with azide, Tween-80, and bromocreosol purple (supplementary table S1*B*, Supplementary Material online) to select for Lactobacillales taxa (Choi et al. 2016). Individual colonies representative of different morphologies were isolated and streaked onto fresh agar (same medium as initial growth but lacking any antibiotics or dyes). A single representative colony was grown in broth of the same medium, visually confirmed as a bacterium by light microscopy (DM5000 B, Leica Microsystems, Buffalo Grove, IL), and stored in 20% glycerol (Sigma, St. Louis, MO) at −80 °C.

### DNA Extraction of Bacterial Isolates

A chunk of frozen glycerol stock was inoculated either onto mMRS or YPD agar and a single colony was obtained to grow in 5 ml broth until turbid (see supplementary table S1*B*, Supplementary Material online, for media). Following Bueno et al. (2019), a 1 ml sample of the cell suspension was centrifuged at 19,000 × g for 5 min and cells were resuspended in 678 μl cell lysis buffer (108 mM Tris–HCl, pH 8.0; 1.5 M NaCl; 21.6 mM EDTA; Sigma) and 16 U proteinase K (Qiagen, Hilden, Germany) with either 30 μl 1 mm diameter glass beads (Scientific Industries, Bohemia, NY) and 250 μl 2.3 mm diameter zirconia beads (BioSpec, Bartlesville, OK) or 200 μl 1 mm diameter glass beads. Samples were homogenized for 35 s at 5.5 m/s with a FastPrep-24 instrument (MP Biomedicals, Santa Ana, CA) and incubated at 56 °C for 2 h. Homogenates were incubated overnight at 37 °C with 35 U RNaseA (Qiagen). DNA was extracted from homogenate with 750 μl phenol:chloroform:isoamyl alcohol (25:24:1; Thermo Fisher Scientific, Waltham, MA) and centrifuged at 19,000 × g for 15 min at 4 °C. To precipitate DNA from 450 μl aqueous layer, 900 μl ethanol and 45 μl 3 M sodium acetate (pH 5.2; Sigma) were added to each sample and incubated overnight at −20 °C. Following centrifugation at 19,000 × g for 15 min at 4 °C, the DNA pellet was washed in 75% ethanol, centrifuged at 19,000 × g for 10 min at 4 °C, air-dried for 10 min, and resuspended in 50 μl nuclease-free water (Ambion, Austin, TX). DNA was stored at −20 °C until PCR amplification and whole-genome sequencing.

### Molecular Identification of Bacteria

Molecular characterization was first performed by Sanger sequencing of bacterial 16S rRNA gene amplicons obtained by PCR with the primers 16SA1 (forward: 5′-AGAGTTTGATCMTGGCTCAG-3′) and 16SB1 (reverse: 5′-TACGGYTACCTTGTTACGACTT-3′) from Fukatsu and Nikoh (1998). Approximately 1 µg DNA template (quantified using Nanodrop; Thermo Fisher Scientific) was added to 0.2 μM primers and 1 U OneTaq 2× Master Mix with Standard Buffer (New England BioLabs, Ipswich, MA). PCR reaction conditions were 94 °C for 30 s, 30 amplification cycles of 94 °C for 30 s, 55.3 °C for 60 s, and 68 °C for 60 s with a final extension for 5 min at 68 °C. PCR products were purified using ExoSAP-IT PCR Clean Up Reagent (Applied Biosystems, Waltham, MA) and submitted for Sanger sequencing (both forward and reverse directions) at Cornell University Genomics Facility using Applied Biosystems 3730xl. Consensus sequences were generated from forward and reverse sequences and taxonomic identity was assigned using BlastN (https://blast.ncbi.nlm.nih.gov/Blast.cgi; last accessed: June 14, 2021) against the NCBI nonredundant nucleotide collection with Geneious Prime 2019.2.1 (Biomatters, Auckland, New Zealand). Bacterial isolates were selected for genome sequencing by maximizing taxonomic, fly replicate, and collection diversity within Enterobacterales, Lactobacillales, and Rhodospirillales.

### Sequencing and Genome Assembly

Genomic DNA (0.2 ng/μl; quantified by Qubit 2.0 fluorimeter; Invitrogen, Waltham, MA) was submitted to Cornell University Genomics Facility for whole-genome shotgun sequencing using an Illumina NextSeq500 Platform with the Nextera XL DNA Library Preparation kit (Illumina, San Diego, CA) to generate 150-bp paired-end reads according to manufacturer’s protocol. Libraries were pooled in equal proportions across three runs and their quality was assessed with a Fragment Analyzer (Advanced Analytical Technologies, Ames, IA). A Blue Pippin device (Sage Science, Beverley, MA) was used for further size-selection of pooled libraries to target fragments ≤800 bp, if required.

Between 1,033,730 and 28,432,172 reads were obtained for 81 bacterial genomes (supplementary table S1*A*, Supplementary Material online). Read quality was assessed using FastQC v0.11.3 (www.bioinformatics.babraham.ac.uk/, last accessed: June 14, 2021) and were trimmed with trimmomatic v0.36 (Bolger et al. 2014). Reads were trimmed on the ends if the quality score was <3 or the terminal base was unidentified (“N”), and sequences were only retained if they had a quality score of ≥15 over a 4-bp moving window and length of 125 bp. Then, SPADES v3.11.1 (Bankevich et al. 2012) was used to assemble reads into contigs (k-mer lengths 21, 33, 55, and 77 were used) following default parameters. The careful option was included for genome polishing. Low k-mer coverage contigs were filtered to reduce contamination following Douglass et al. (2019); see supplementary table S1*A*, Supplementary Material online, for cutoffs applied to each genome. SSPACE v3.0 (Boetzer et al. 2011) was used for contig extension and scaffolding following default parameters with a minimum 100 bp contig length (insert size was estimated from subsampling 1,000,000 reads). Genome assembly statistics were obtained using Quast v4.6.3 (Gurevich et al. 2013) with contigs less than 500 bp removed. To assess average sequence depth, reads were mapped to final contigs using Bowtie2 v2.2.6 (Langmead and Salzberg 2012) following default parameters and the SAMtools v0.1.19 (Li et al. 2009) depth function. ConEst16S (Lee et al. 2017) was used to identify bacterial contamination when more than one 16S rRNA gene was detected for a genome (supplementary table S1*A*, Supplementary Material online); none of the genomes were found to have bacterial contamination.

### Genome Annotation

Genomes were annotated using the RASTtk pipeline on RAST server with error correcting (Overbeek et al. 2014; Brettin et al. 2015). Specifically, the settings were set to automatically fix errors and fix frameshifts. For analysis of primary metabolism genes, the following RAST categories were extracted: amino acids and derivatives; carbohydrates; cofactors, vitamins, prosthetic groups and pigments; fatty acids, lipids, and isoprenoids; nitrogen metabolism; and nucleosides and nucleotides. The RAST subsystems associated with secondary metabolism (cyanate hydrolysis, hopanes, polyhydroxybutyrate metabolism, nitrilase, and nitrosative stress), and the nucleosides and nucleotides subcategories detoxification and “no subcategory” were removed to retain the main nucleotide biosynthesis, conversion, and degradation genes. For the selected primary metabolism functions, all genes in the “no subcategory” subsystems were combined into an “other” subcategory for each RAST category (apart from nucleoside and nucleotide category). This final set of functions largely focuses on primary metabolism traits, although some of these genes may, additionally or alternatively, encode functions that contribute to secondary metabolism (due to gene duplication or as by-products of primary metabolism). For analyses, each RAST role (or gene function) was counted once, although there may be several genes (or RAST features) that are annotated with each function due to gene duplication events, fragmented genes across contigs, or nonspecific annotations of function. Due to the large variation in total CDSs for each strain (supplementary table S1*A*, Supplementary Material online), relative counts were generated for the number of functions found in each RAST subcategory (scaled to the total number of primary metabolism-related functions). The full data set (including categorization prior to extraction) is provided in supplementary data set S3, Supplementary Material online. The GenBank flat file of publicly available genomes for other wild *Drosophila*-associated bacteria was downloaded from NCBI (supplementary table S1*C*, Supplementary Material online) and were reannotated using RAST to obtain functional trait data. For pangenome analysis, metabolic genes were extracted using a custom R script for species with more than four strains and were reannotated using PROKKA v1.14.6 (Seemann 2014). For orthogroup analysis, Eggnog Mapper v2 (Huerta-Cepas et al. 2019) was implemented to annotate representative sequences from each orthogroup as a general annotation, whereas a custom R script was used to associate RAST metabolic functions with metabolism-related orthogroups for statistical analyses.

### Orthologous Group Gene Clustering and Pangenome Analysis

OrthoFinder v2.4.0 (Emms and Kelly 2015, 2019) was implemented to cluster protein-coding sequences into orthogroups for all *Drosophila-*associated bacteria with default settings. Several reference genomes (supplementary table S1*C*, Supplementary Material online) were included in the initial run for analysis of species tree to identify single ortholog genes shared across all taxa for phylogenomic analysis. For metabolism-related clusters, reference genomes were pruned from orthogroup list, and a custom R script was used to extract orthogroups containing relevant metabolic functions (based on RAST annotations). HMMER v3.3.1 was used to identify representative amino acid sequences for each orthogroup using “hmmbuild” and “hmmsearch” functions (HMMER: hmmer.org, last accessed: June 14, 2021) for Eggnog Mapper annotation. In addition, Roary v3.13.0 (Page et al. 2015) was used to assess variation in metabolic repertoire of prevalent species using PROKKA annotations. The pangenome distribution index was calculated as a corrected proportion of the number of core genes (subtracting the accessory gene count from the core gene count of each strain) scaled to the total number of genes found in the pangenome.

### Phylogenetic and Phylogenomic Reconstructions

Sequences for single-gene and multilocus phylogenies were aligned using MUSCLE (Edgar 2004) with default settings in Geneious Prime and phylogenetically informative sites were selected with GBlocks v0.91b (Castresana 2000) using less stringent options (b1–b5 settings: 0.5, 0.55, 8, 5, half). Maximum likelihood phylogenies were generated using IQ-TREE v1.6.12 (Nguyen et al. 2015) with model of evolution chosen by lowest BIC score with ModelFinder (Kalyaanamoorthy et al. 2017). Bootstrap replicates (10,000 replicates with ultrafast bootstrap approximation method) were performed to identify node support using UFBoot2 (Hoang et al. 2018). For the phylogenomic reconstruction, single orthologous gene clusters identified using OrthoFinder (52 amino acid sequences, see supplementary table S7, Supplementary Material online) were concatenated with SequenceMatrix v1.8 (Vaidya et al. 2011) for a partitioned model (proportional branch lengths implemented) with IQ-TREE (Chernomor et al. 2016).

Species boundaries of sampled taxa were determined using a 95% average nucleotide identity (ANI) score threshold using JSpecies v1.2.1 (Richter and Rosselló-Móra 2009) with MuMmer v3.23 (Kurtz et al. 2004) at default settings. Taxa identities were confirmed by comparing each strain to related genomes (type specimens accessed from NCBI) and a BlastN search for genome extracted 16S rRNA gene sequences (supplementary table S1*A*, Supplementary Material online). Individual phylogenies for each bacterial order were drawn by extracting each clade from the entire reconstruction using the packages ape v5.4 (Paradis and Schliep 2019) and ggtree v2.2.4 (Yu et al. 2017) with *Vibrio cholerae, Bacillus subtilis*, and Rhodospirillaceae spp. (*Magnetospirillum magneticum* and *Rhodospirillum rubrum*) used to root phylogenies of the Enterobacterales, Lactobacillales, and Rhodospirillales, respectively.

### Statistics

All analyses were performed using R v4.0.2 (R Core Team 2018) with a significance α threshold of 0.05. All statistical analyses were performed using *Drosophila-*associated strains and did not include the reference strains (supplementary table S1*C*, Supplementary Material online) used for phylogenetic analyses (taxa excluded: *B. subtilis*, *Enterococcus faecalis*, *Streptococcus pyogenes*, *Haemophilus influenzae*, *Pasturella multocida*, *Granulibacter bethesdensis*, *M. magneticum*, *R. rubrum*, and *V. cholerae*). Genome features (CDS, genome size, metabolic function count, and GC content) were assessed for phylogenetic signal using two different univariate methods. First, Pagel’s λ was imputed to determine whether genomic features could be explained by phylogenetic relatedness as compared with a Brownian motion model of evolution using a likelihood ratio test (null hypothesis: λ = 0 or completely random) with the package phytools v0.7.47 (Revell 2012). Then, an analysis of variance (ANOVA) was implemented to assess the categorical effect of taxonomy on genomic features with patristic distance (sum of branch lengths from root tip) as a covariate using the car package v3.0.8 (Fox and Weisberg 2019), except a logistic regression (quasibinomial distribution with logit link) was implemented to analyze GC content with a Wald’s χ^2^ test for the omnibus test. Patristic distance was calculated with the “distRoot” function from the package adephylo v1.1.11 (Jombart et al. 2010) using a *Lactococcus lactis* Bpl1 rooted tree (note: reference strains were removed for statistical analyses). Normality and homoscedasticity of residuals were visually assessed for each model. For all models, genome size and CDS were log_10_-transformed.

Several multivariate methods were implemented to identify relationships among bacterial metabolic traits and orthogroups. First, RAST subcategories were visualized by bacterial strain using PCoA with Bray–Curtis dissimilarities on relative counts (proportions were based on total number of function counts in selected RAST subcategories related to primary metabolism pathways to reduce the variation in counts; see heatmap of relative counts in supplementary fig. S8, Supplementary Material online) using the “capscale” function in the vegan package v2.5.6 (Oksanen et al. 2019). Orthogroup incidence was visualized with a PCoA using a Jaccard similarity coefficient for presence–absence data. Second, a permutational multivariate analysis of variance (PERMANOVA) was performed with the “adonis” function to determine whether metabolic traits and orthogroup incidence varied by bacterial taxonomy with 999 permutations and Bray–Curtis dissimilarities on relative count data or Jaccard similarity coefficient for presence–absence data. A post hoc pairwise PERMANOVA was implemented using the “adonis.pair” function from the EcolUtils package v0.1 (Salazar 2020) with 999 permutations and Benjamini–Hochberg false discovery rate *P* value adjustment method (FDR). Then, a Ward’s linkage agglomerative hierarchical cluster was applied to relative count data with Bray–Curtis dissimilarities to generate a dendrogram by bacterial strains. The pvclust package v2.2.0 (Suzuki et al. 2019) was implemented to identify significant clusters in the hierarchical cluster with approximately unbiased *P* values and bootstrap probability support values (*n* = 10,000). Finally, PCoA of the metabolism-related orthogroups were correlated with the PCoA of all orthogroups using a Procrustean randomization test (999 permutations) in the vegan package with the function “protest.”

Correlation between dendrograms was determined using two metrics. First, normalized Robinson–Foulds (nRF) metric was calculated using the phangorn package v2.5.5 (Schliep 2011) to test for congruence between dendrogram topologies. nRF values are bounded between 0 and 1, corresponding to complete congruence to incongruence. Then, a Mantel test was performed to associate two distance matrices using Spearman’s rank correlation with 999 permutations using vegan. For phylogenies, cophenetic distances (pairwise sum of branch lengths) were calculated using the “cophenetic.phylo” function in ape. Bray–Curtis dissimilarities were used for the relative function counts. Tanglegrams were generated using the dendextend package v1.13.4 (Galili 2015) with the “step2side” aligner.

For the analysis of prevalent species, represented in at least four flies and comprising >4 strains, several methods were used to compare pangenome distribution and functional content. Differences in the pangenome distribution index were examined with a beta regression using the betareg package v3.1.3 (Cribari-Neto and Zeileis 2010). A likelihood ratio test was used to assess the effect of species by comparing the regression to an intercept-only model with the package lmtest v0.9.37 (Zeileis and Hothorn 2002) and a post hoc Tukey’s test was implemented with the emmeans package. Pearson’s product-moment correlation coefficient was used to assess linear association between strain diversity and pangenome distribution gene count. Strain diversity was scored in two ways: first with Shannon’s entropy on the concatenated amino acid sequence alignment used in the phylogenomics analysis with the Bio3d package (Grant et al. 2006) and then nucleotide diversity was obtained for 16S rRNA gene alignment with the pegas package (Paradis 2010). A two-sided Fisher’s exact test was used to compare orthogroup incidence between species, whereas a one-sided Fisher’s exact test was used for enrichment of subsystems in the accessory genome compared with the core genome of each species. The odds ratios (OR) were calculated based of the function count of a given subsystem in the accessory genome relative to the rest of the function counts in the core genome. FDR method was used to adjust for multiple Fisher’s exact tests.

## Supplementary Material

Supplementary data are available at *Genome Biology and Evolution* online.

## Supplementary Material

evab127_Supplementary_Data
