# Supplementary material for: Genome-Inferred Correspondence between Phylogeny and Metabolic Traits in the Wild Drosophila Gut Microbiome
Source: Genome Biol Evol. 2021 Jun 3;13(8):evab127. doi: 10.1093/gbe/evab127 (PMC8358223; doi:10.1093/gbe/evab127)
Supplement: evab127_Supplementary_Data [file evab127_supplementary_data.zip › Supplemental list of files.docx]

**Supplemental files**

**Table S1. List of bacteria used in comparative genomics analysis** (A) Summary of genome sequencing and strain isolation for new *Drosophila*-associated bacteria (B) Recipes for media used to isolate new bacterial strains (C) Publicly available genomes used in analyses.

**Table S2. Percent identity among 16S rRNA gene nucleotide sequences** (A) Enterobacterales (B) Lactobacillales (C) Rhodospirillales

**Table S3. *Post hoc* pairwise comparisons for PERMANOVAs** (A) Comparisons among orders for RAST normalized function counts (Figure 3) (B) Comparisons among orders for orthogroup incidence for all taxa (Figure S5A) (C) Comparisons among orders for metabolic orthogroup incidence for all taxa (Figure S5B)

**Table S4. Annotations and incidence of orthogroups** (A) Eggnog mapper annotations for all orthogroups (B) Full dataset for orthogroups with 3 or more genomes represented (C) Metabolism-related orthogroups with 3 or more genomes represented

**Table S5. Metabolic orthogroups of prevalent species and Fisher's exact test results** (A) Full set of orthogroups examined (B) Top orthogroups identified

**Table S6. Subsystem enrichment analysis of prevalent species** (A) *Acetobacter thailandicus* (B) *Gluconobacter cerinus* (C) *Gluconobacter kondonii* (D) *Levilactobacillus brevis* (E) *Lactiplantibacillus plantarum* (F) *Providencia rettgeri* (G) *Tatumella* sp. (H) Gene functions among top subsystems and distribution across pangenomes

**Table S7. Gene annotation and model selection for 52 amino acid sequences used in phylogenomic reconstruction of *Drosophila*-associated bacteria**

**Fig. S1. Species boundary delineation among *Drosophila*-associated strains** (A) Enterobacterales (B) Lactobacillales (C) Rhodospirillales

**Fig. S2. 16S rRNA gene phylogeny** (A) Enterobacterales clade extraction (B) Lactobacillales clade extraction (C) Rhodospirillales clade extraction.

**Fig. S3. Principal coordinates analysis (PCoA) of metabolic functions by genus**

**Fig. S4. Mapping function onto phylogeny** (A) Correlation between multi-locus species phylogeny and Bray-Curtis dissimilarity based hierarchical cluster of relative metabolic function counts (B) Correlation between 16S rRNA gene phylogeny and Bray-Curtis dissimilarity based hierarchical cluster of relative metabolic function counts

**Fig. S5. Principal coordinates analysis (PCoA) of orthogroup incidence** (A) Representation of all 13,170 orthogroups with at least three genomes present in each. (B) Visualization of the 1,055 metabolism-related orthogroups extracted from the full dataset

**Fig. S6. Strain diversity among metabolic pangenomes and gene distribution** (A) Rarefaction curves generated from Roary analysis using log-log linear model (B) Correlation between pangenome distribution for each species with residue diversity (Shannon’s entropy calculated from phylogenomic amino acid sequence alignment) and (C) nucleotide diversity among 16S rRNA gene alignments

**Fig. S7. Association of metabolic function with composite representation of orthogroup incidence among prevalent species** (A) PC1 and (B) PC2 from PCoA in Figure 4.

**Fig. S8. Relative abundance of RAST functional categories.**

**Dataset S1. Genomic features and phylogenetic distance of *Drosophila*-associated strains**

**Dataset S2. Pangenome distribution index, gene count, and strain diversity of prevalent species**

**Dataset S3. RAST functional annotation counts** (A) All subcategories found in all 96 taxa (B) All subcategories related to primary metabolism found in all 96 taxa

**Dataset S4. Multi-locus phylogeny sequence alignment**

**Dataset S5. 16S rRNA gene phylogeny sequence alignment**
