# Supplementary material for: Genome-Inferred Correspondence between Phylogeny and Metabolic Traits in the Wild Drosophila Gut Microbiome
Source: Genome Biol Evol. 2021 Jun 3;13(8):evab127. doi: 10.1093/gbe/evab127 (PMC8358223; doi:10.1093/gbe/evab127)
Supplement: evab127_Supplementary_Data [file evab127_supplementary_data.zip › Supplemental_Figures.pdf]

A)

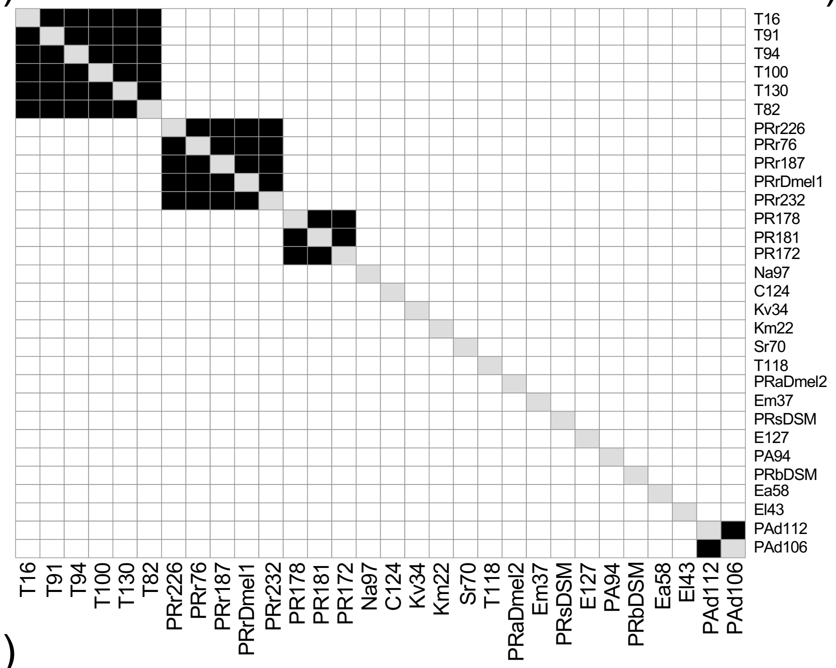

B)

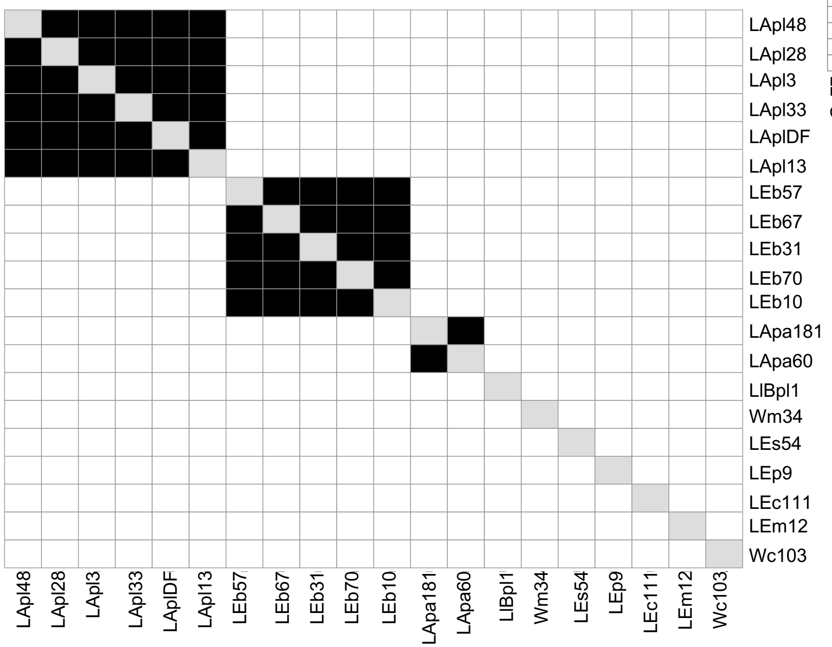

C)

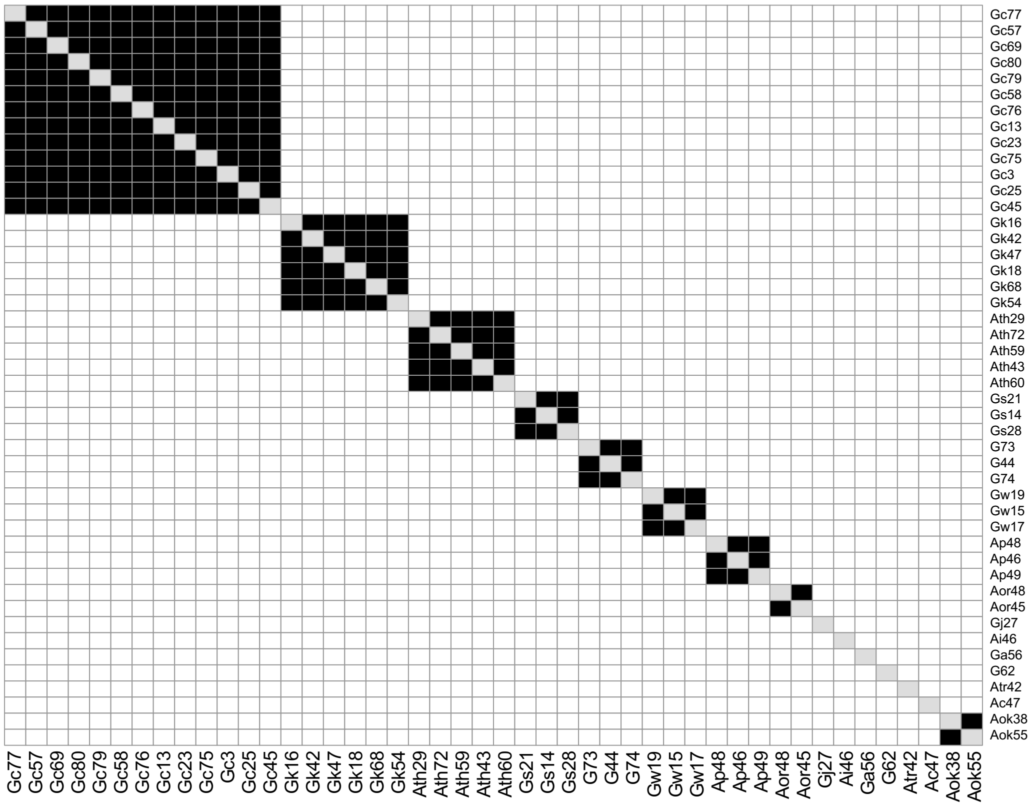

Fig. S1. Species boundary delineation among *Drosophila*-associated strains. (A) Enterobacterales. (B) Lactobacillales. (C) Rhodospirillales. Black cells of heatmap correspond to pairwise average nucleotide identity (ANI) score of 95% or greater, while white cells indicate scores < 95%.

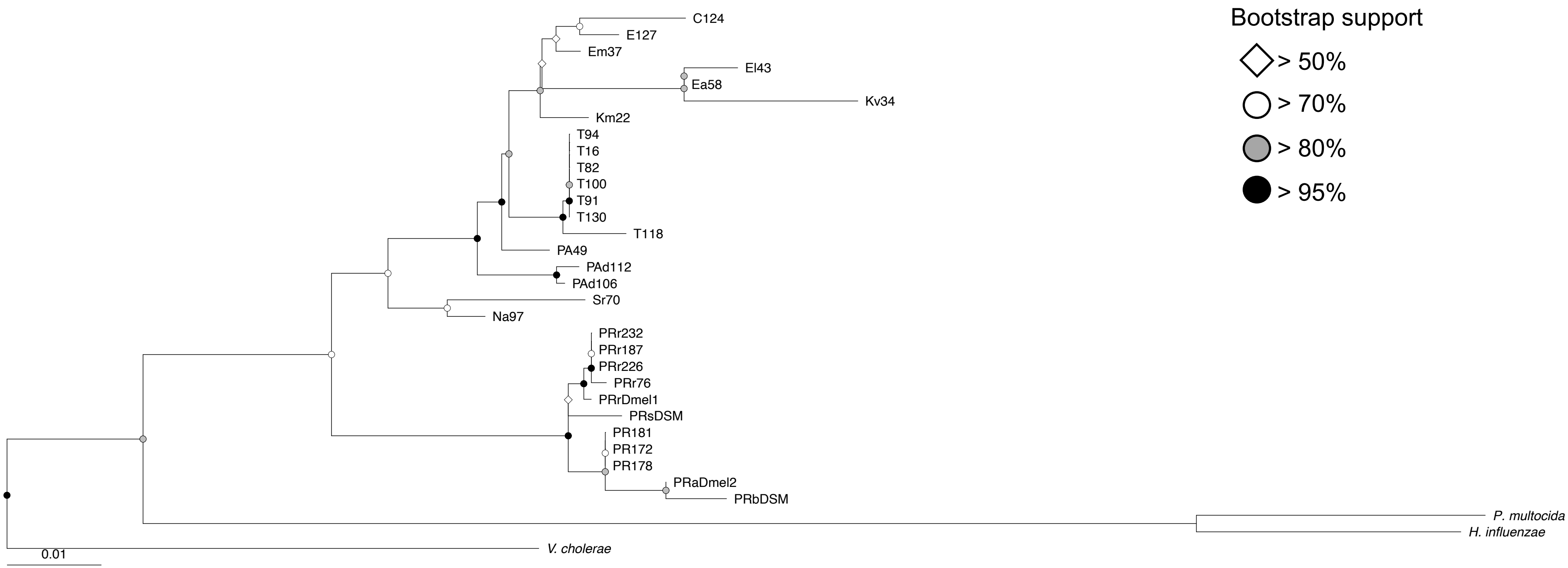

Fig. S2. 16S rRNA gene phylogeny. (A) Enterobacterales clade extraction. *V. cholerae, H. influenzae,* and *P. multocida* are used as reference strains for studied taxa. Maximum likelihood tree was generated with a TIM3+F+I+G4 model of evolution (length = 1441 bp) with 10,000 ultrafast bootstrap replicates. Tree is scaled by number of nucleotide substitutions per site. Sequence alignment is provided in Dataset S5.

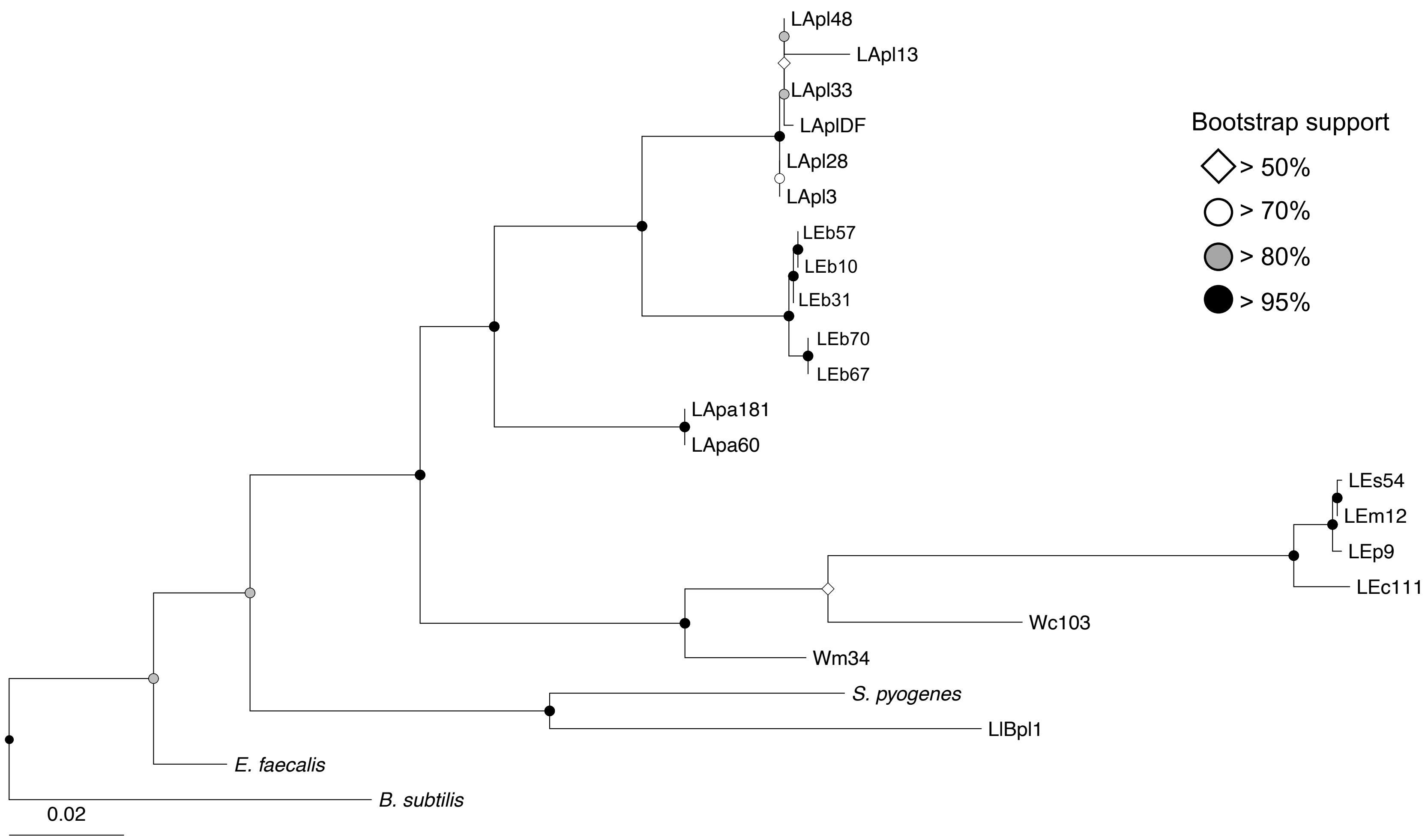

Fig. S2. 16S rRNA gene phylogeny. (B) Lactobacillales clade extraction. *B. subtilis, E. faecalis,* and *S. pyogenes* are used as reference strains for studied taxa. Maximum likelihood tree was generated with a TIM3+F+I+G4 model of evolution (length = 1441 bp) with 10,000 ultrafast bootstrap replicates. Tree is scaled by number of nucleotide substitutions per site. Sequence alignment is provided in Dataset S5.

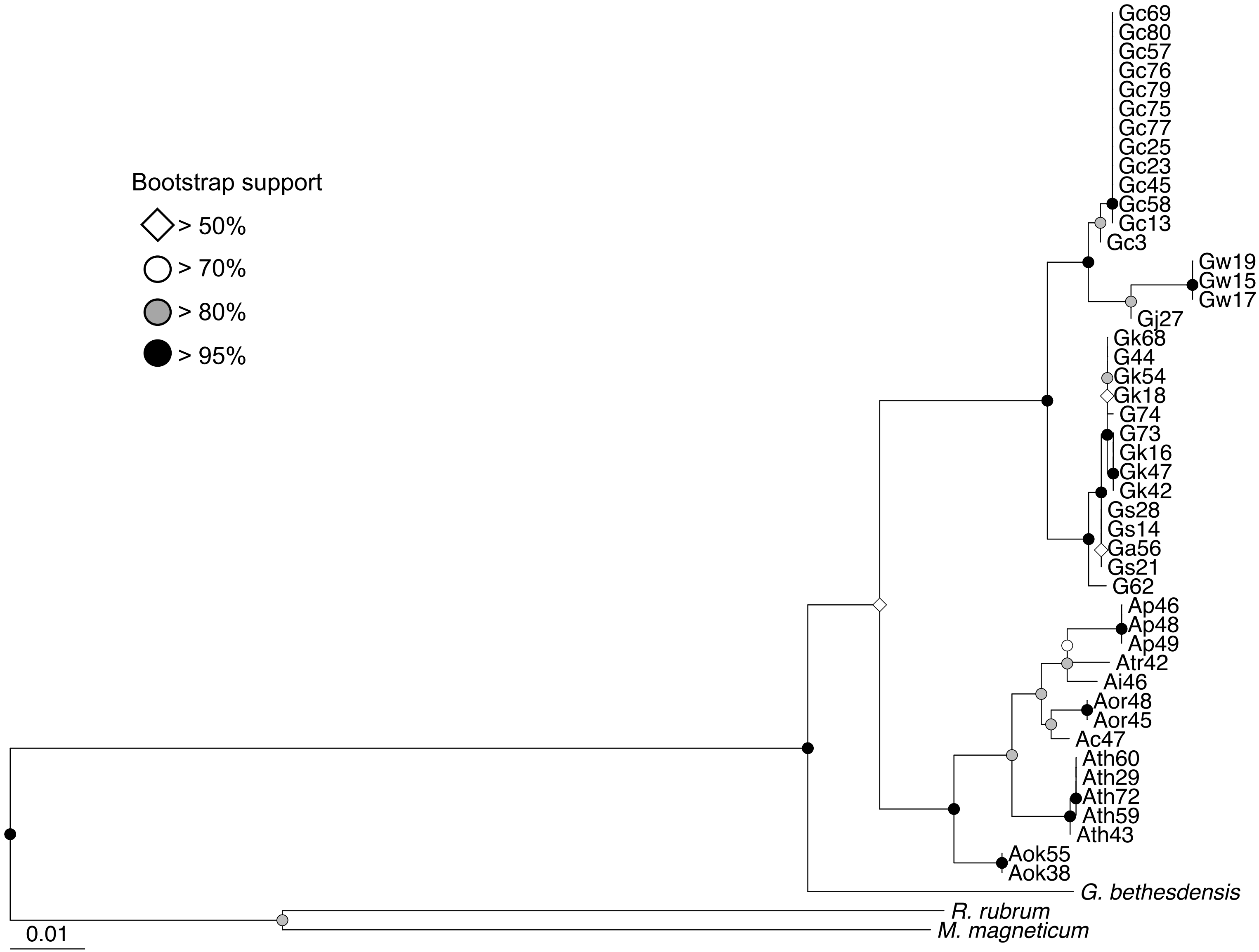

Fig. S2. 16S rRNA gene phylogeny. (C) Rhodospirillales clade extraction. *M. magneticum, R. rubrum,* and *G. bethesdensis* are used as reference strains for studied taxa. Maximum likelihood tree was generated with a TIM3+F+I+G4 model of evolution (length = 1441 bp) with 10,000 ultrafast bootstrap replicates. Tree is scaled by number of nucleotide substitutions per site. Sequence alignment is provided in Dataset S5.

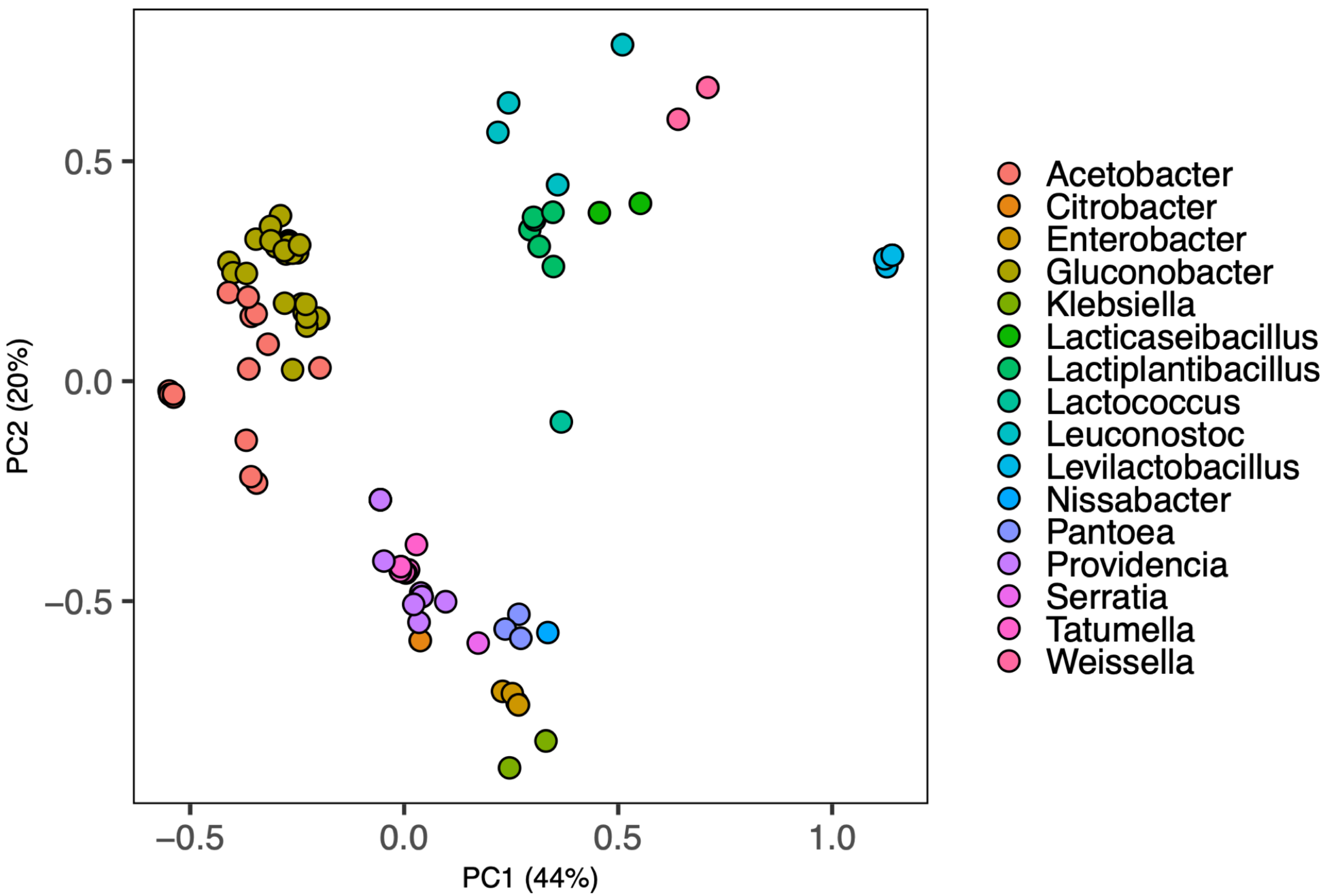

Fig. S3. Principal coordinates anlaysis (PCoA) of metabolic functions by genus. All points represent composite of metabolic functional traits for each genome analyzed with colors corresponding to genus-level taxonomy. The percentages on each axis correspond to the amount of variation explained.

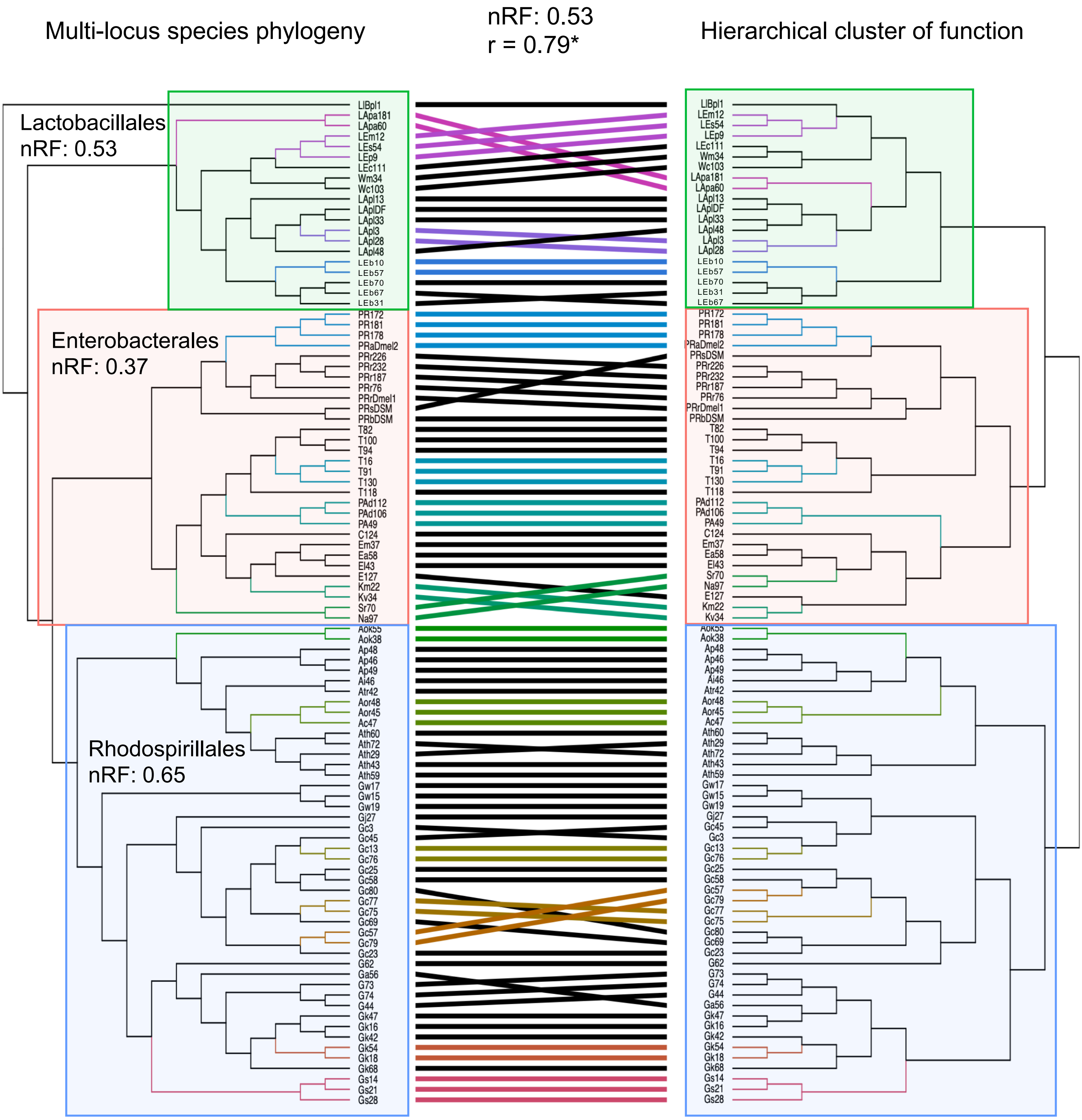

Fig. S4. Mapping function onto phylogeny. (A) Correlation between multi-locus species phylogeny and Bray-Curtis dissimilarity based hierarchical cluster of relative metabolic function counts. The normalized Robinson-Foulds index (nRF) and Mantel's test (based on Spearman's rank correlation) for the entire tanglegram are reported at the top. Each bacterial order is indicated by shaded box with the specific nRF obtained for each order. The tips of each dendrogram are connected by either black or colored line; the latter represents subtrees within each dendrogram that have the same topology. * represents p = 0.001.

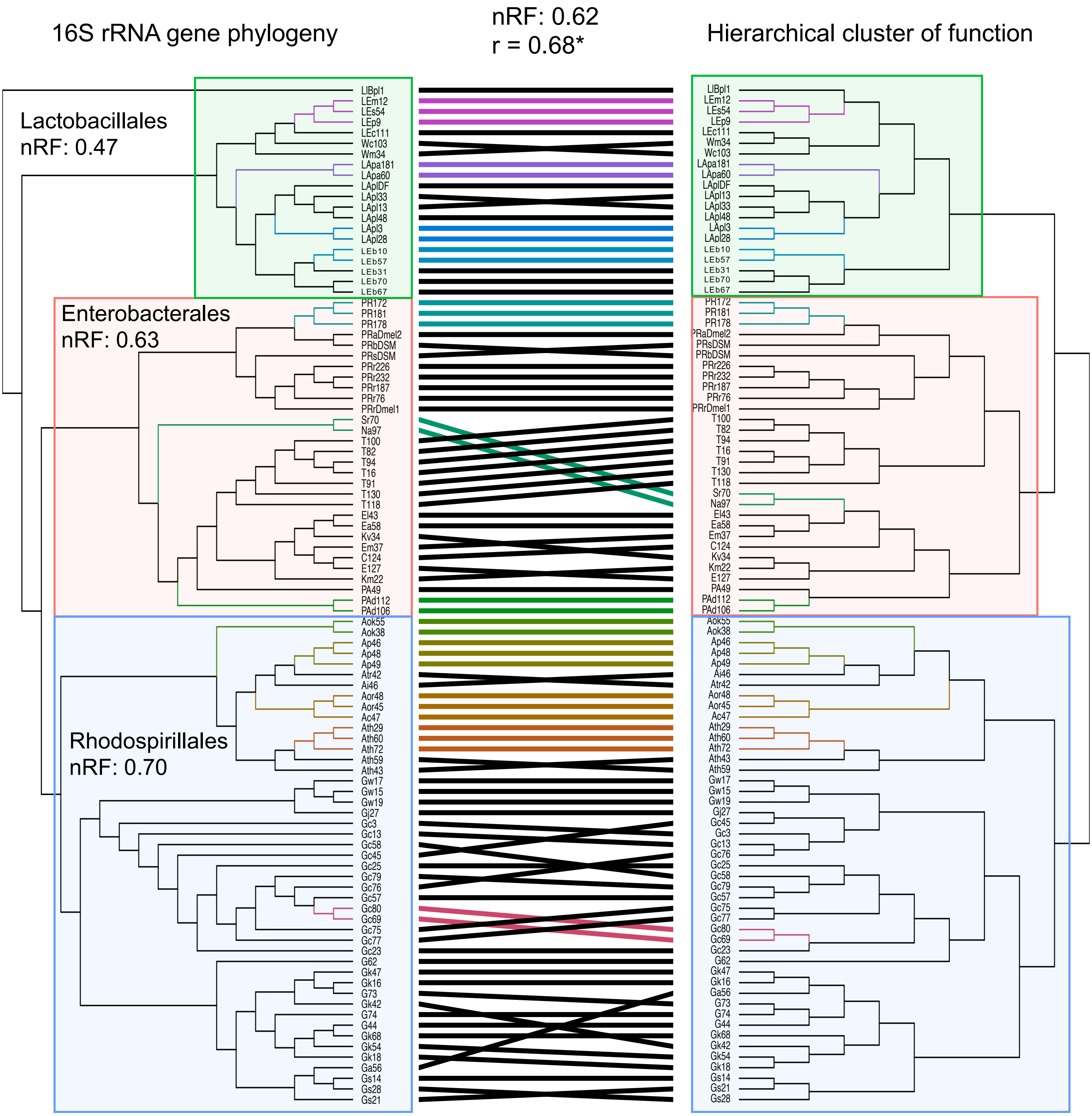

Fig. S4. Mapping function onto phylogeny. (B) Correlation between 16S rRNA gene phylogeny and Bray-Curtis dissimilarity based hierarchical cluster of relative metabolic function counts. The normalized Robinson-Foulds index (nRF) and Mantel's test (based on Spearman's rank correlation) for the entire tanglegram are reported at the top. Each bacterial order is indicated by shaded box with the specific nRF obtained for each order. The tips of each dendrogram are connected by either black or colored line; the latter represents subtrees within each dendrogram that have the same topology. * represents p = 0.001.

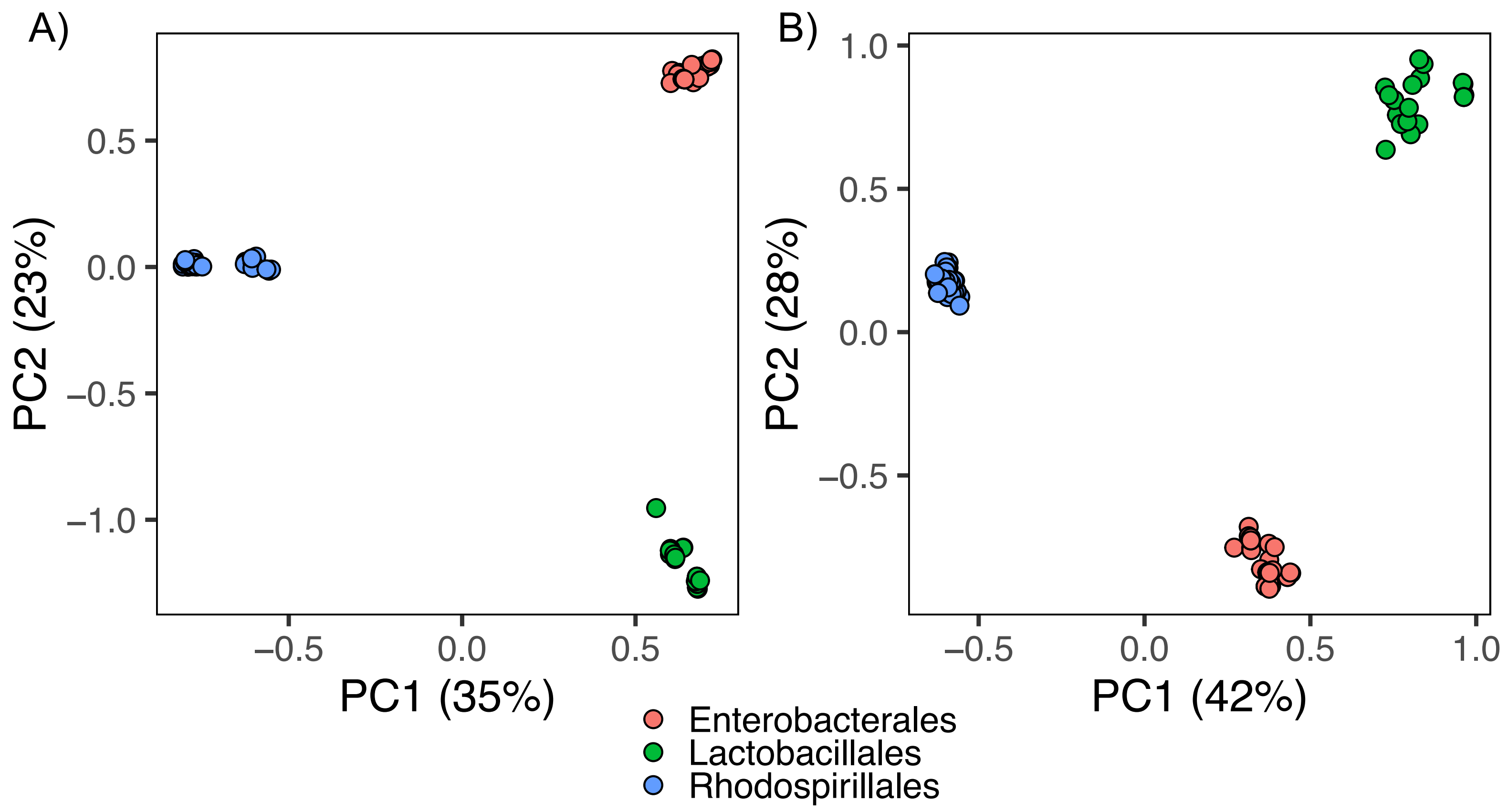

Fig. S5. Principal coordinates analysis (PCoA) of orthogroup incidence. (A) Representation of all 13,170 orthogroups with at least three genomes present in each. (B) Visualization of the 1,055 metabolism-related orthogroups extracted from the full dataset. All points represent composite of orthogroup incidence for each genome analyzed with colors corresponding to order-level taxonomy. The percentages on each axis correspond to the amount of variation explained. Data are provided in Table S5.

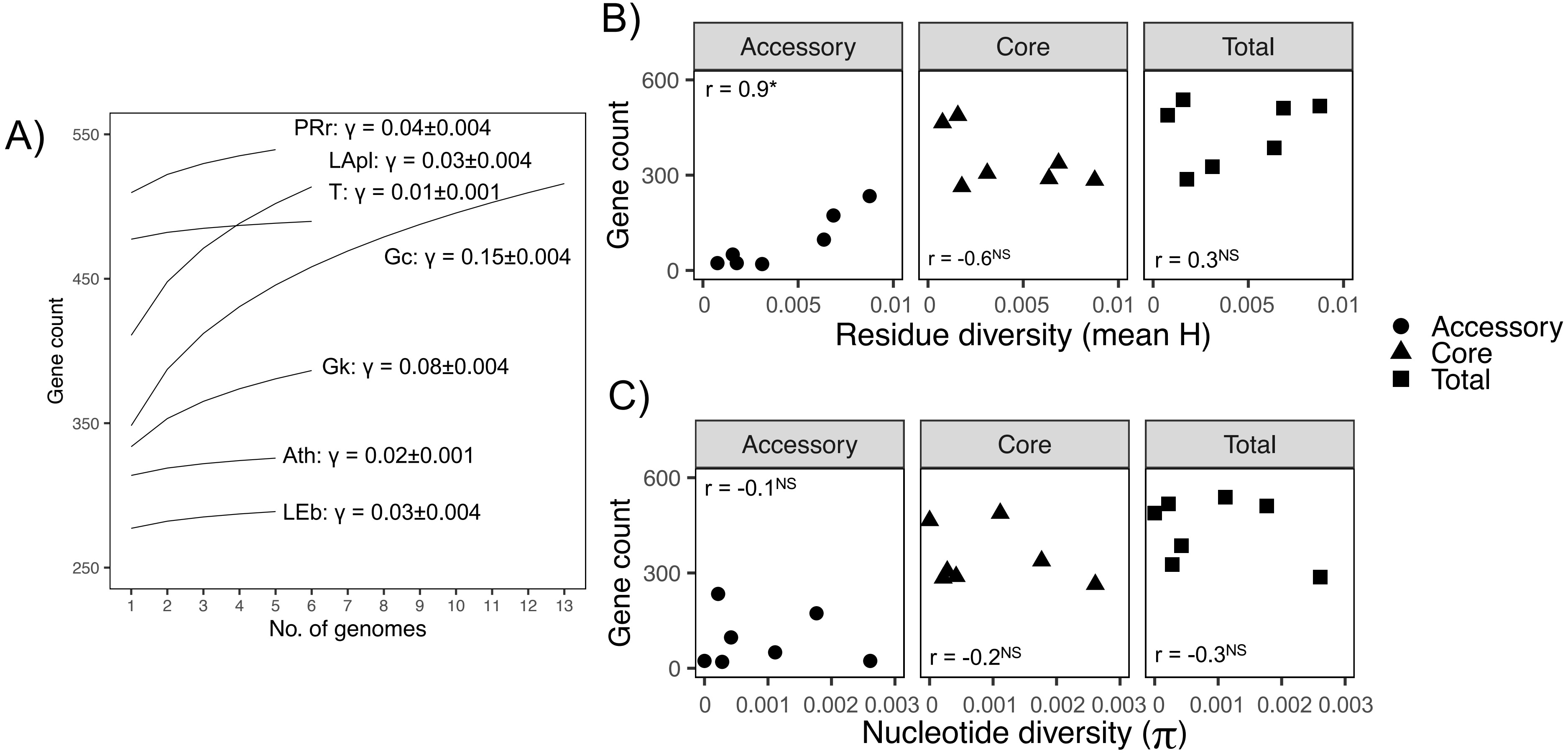

Fig. S6. Strain diversity among metabolic pangenomes and gene distribution. (A) Rarefaction curves generated from Roary analysis using log-log linear model. (B) Correlation between pangenome distribution for each species with residue diversity (Shannon's entropy calculated from phylogenomic amino acid sequence alignment) and (C) nucleotide diversity among 16S rRNA gene alignments. γ in panel A represents the slope and standard error from the log-log linear model, which indicates an "open" pangenome for values < 1. For panels B and C, Pearson's correlation statistic is show. NS = not significant; * p = 0.003. Species identifiers: Ath = *A. thailandicus*, Gc = *G. cerinus*, Gk = *G. kondonii*, LEb = *Le. brevis*, LApl = *La. plantarum*, PRr = *P. rettgeri*, and T = *Tatumella* sp. Data are provided in Dataset 3.

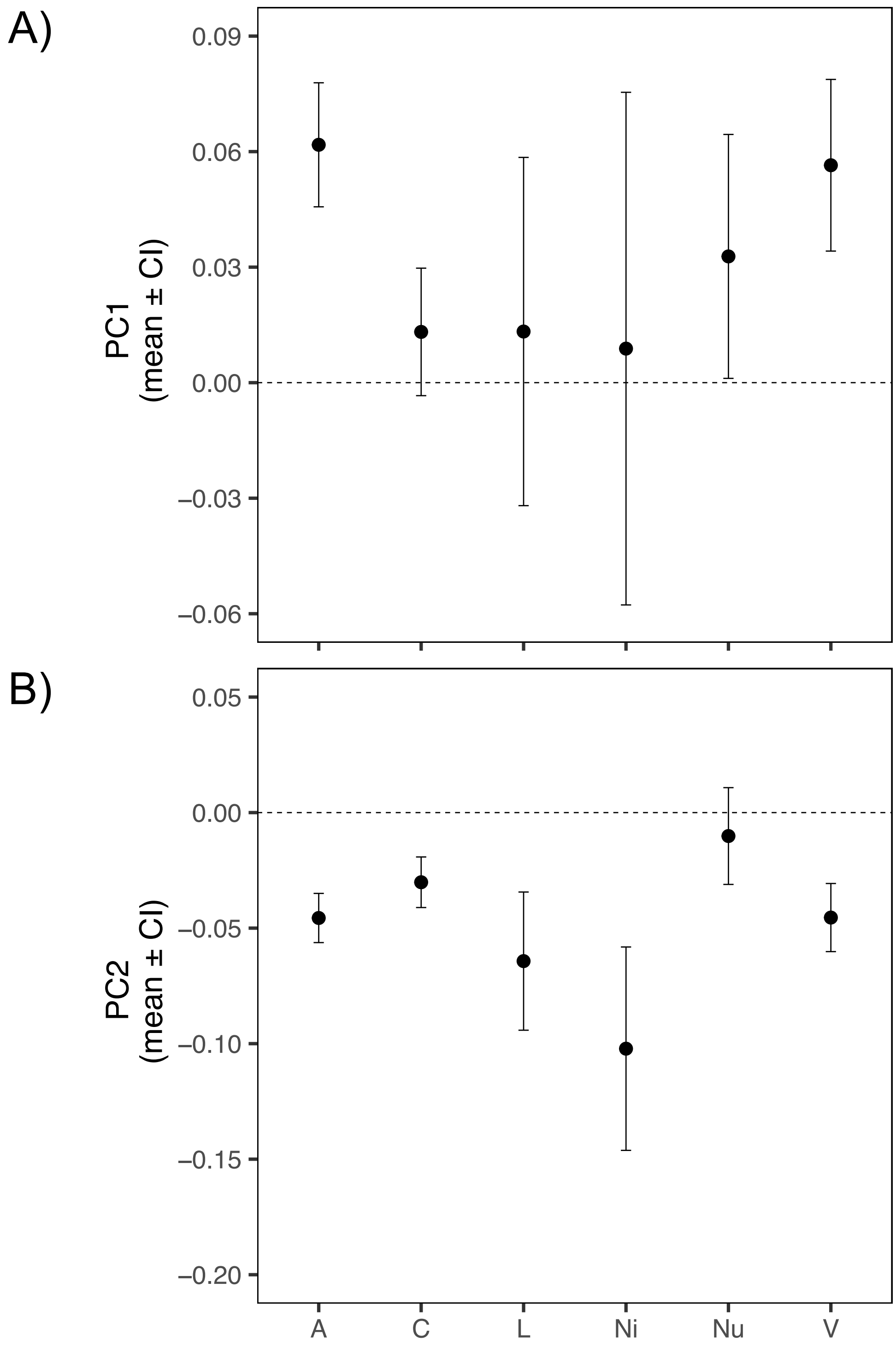

Fig. S7. Association of metabolic function with composite representation of orthogroup incidence among prevalent species. (A) PC1 and (B) PC2 from PCoA in Figure 4. The means and 95% confidence interval are plotted. Non-overlapping confidence intervals indicate significant association with function and taxa deviations from PCoA center (0,0). RAST categories: A = amino acids and derivatives, C = Carbohydrates, V = cofactors, vitamins, prosthetic groups, and pigments, L = fatty acids, lipids, and isoprenoids, Ni = nitrogen metabolism, and Nu = nucleosides and nucleotides.

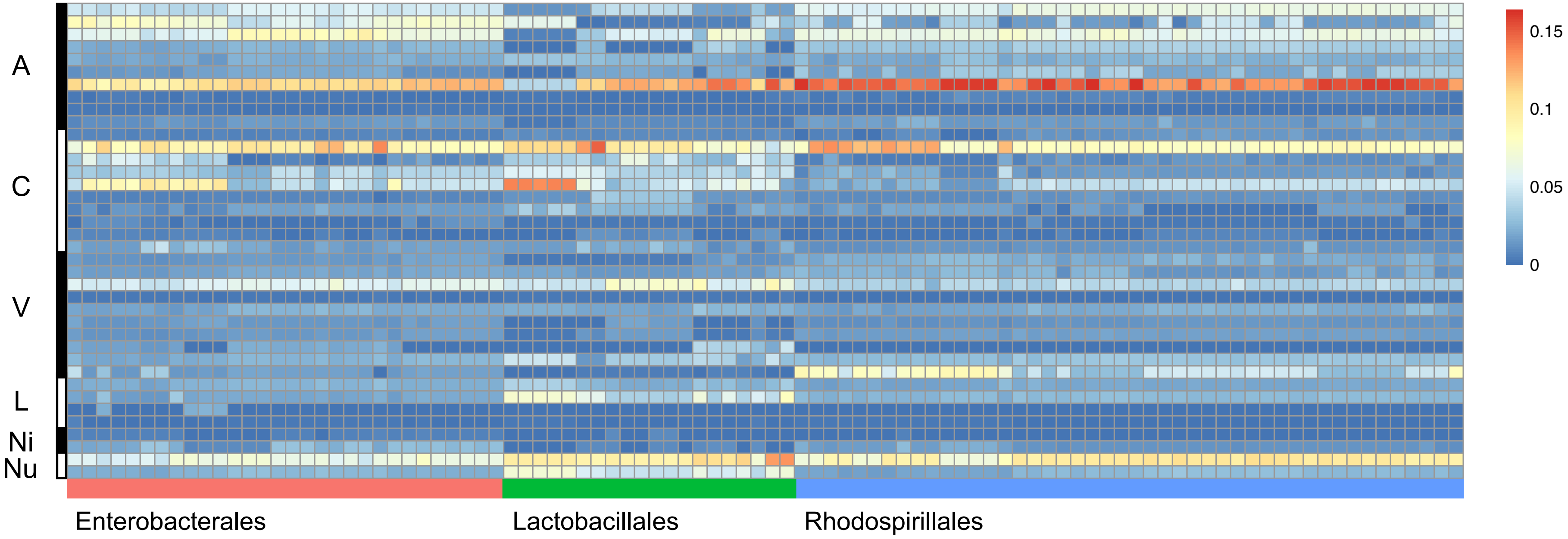

Fig. S8. Relative abundance of RAST functional counts. Data was transformed to relative counts of RAST functions for each genome in order to adjust all the counts to similar scales. Many of the functions exhibit similar patterns as found in Fig. 3A with distinct differences among each order.
